# A posteriori error estimates for mixed-dimensional Darcy flow using non-matching grids

**DOI:** 10.1007/s10543-026-01158-0

**Published:** 2026-10-26

**Authors:** Jhabriel Varela, Christian E. Schaerer, Eirik Keilegavlen, Inga Berre

**Affiliations:** 1 Polytechnic School, National University of Asunción, Campus de la UNA, P.O. Box 2111 SL, San Lorenzo, Paraguay; 2 Polytechnic University Taiwan-Paraguay, Sebastián Gaboto - Comando de Comunicaciones del Ejército, P.O. Box 1018 AS, Asunción, Paraguay; 3 Center for Modeling of Coupled Subsurface Dynamics, Department of Mathematics, University of Bergen, Allégaten 41, P.O. Box 7800 N-5020, Bergen, Norway

**Keywords:** A posteriori error estimates, Mixed-dimensional elliptic equations, Non-matching grids, Single phase flow in fractured porous media, 65N15, 65N50, 76S05

## Abstract

In this article, we extend the *a posteriori* error estimates for hierarchical mixed-dimensional elliptic equations developed in [Varela et al., *J. Numer. Math.*, 31 (2023), pp. 247–280] to the setting of non-matching mixed-dimensional grids. The extension is achieved by introducing transfer grids between the planar subdomain and interface grids, together with stable discrete projection operators for primal (potential) and dual (flux) variables and grid mismatch operators. The proposed estimators remain guaranteed, reliable and fully computable for locally mass-conservative approximations. Numerical experiments, including three-dimensional problems based on community benchmarks for incompressible Darcy flow in fractured porous media, demonstrate reliable performance of the estimators for the non-matching grids and effectivity that is comparable to the estimators previously obtained for matching grids.

## Introduction

The scalar hierarchical mixed-dimensional (mD) elliptic equation extends the standard Poisson equation by incorporating the coupling between domains of co-dimension one [1]. This framework is particularly well suited for modeling flow processes in fractured porous media [2, 3], where the rock matrix is represented as a 3D host domain, fractures as embedded 2D planar surfaces, fracture intersections as 1D line segments, and their intersections as 0D points. Over the past two decades, this model has been extensively studied, and a variety of techniques have been proposed to solve it; we refer the reader to the review articles [4, 5] and community benchmarks [6, 7] for comprehensive overviews. Notably, the model has been recently validated experimentally [8], further establishing its relevance and applicability.

Given that mD problems naturally span subdomains of different dimensions, it is both geometrically and computationally advantageous to discretize each subdomain with an independent grid, avoiding the constraints of matching grids. Previous works that have considered modeling and discretization of flow in fractured porous media on non-matching grids include [9–13]. In parallel, *a posteriori* error estimates were also developed for problems in mixed dimensions. For instance, [14] in the context of multi-scale mortar methods, [15–19] for Darcy flow in fractured media, and more recently [20] for hierarchical mD elliptic problems in the context of Darcy flow in networks of planar fractures. To our knowledge, however, no existing estimators cover the fully general mD setting—incorporating intersecting and fully embedded subdomains—on non-matching grids.

**Fig. 1:**
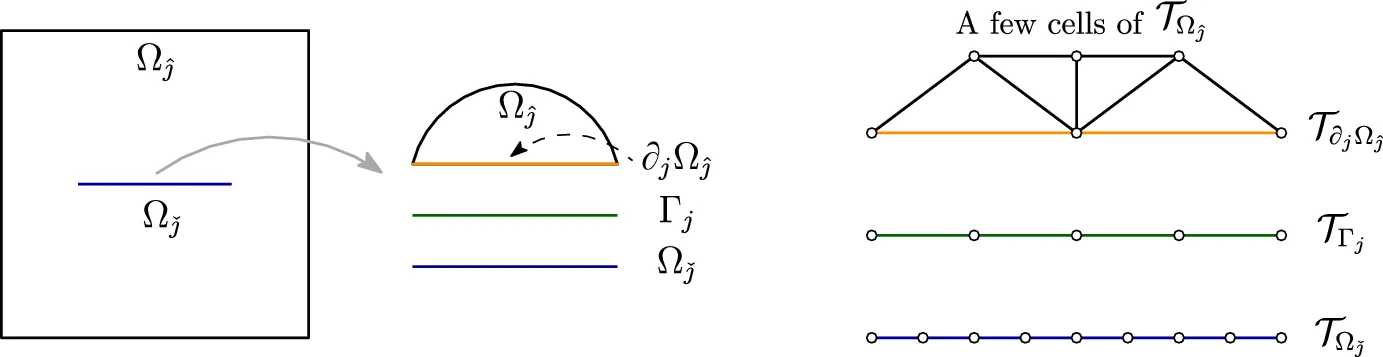
Mixed-dimensional geometric setting. Left: A fracture $\Omega _{{\check{\jmath }}}$ fully embedded in the matrix $\Omega _{{\hat{\jmath }}}$. A close-up of the upper side of the fracture shows the interface $\Gamma _j$ coupling $\Omega _{{\check{\jmath }}}$ and $\Omega _{{\hat{\jmath }}}$ through its internal boundary $\partial _j\Omega _{{\hat{\jmath }}}$. There exists a similar coupling on the lower side of the fracture, not shown in the figures. Right: A non-matching coupling between $\mathcal {T}_{\partial _{j}\Omega _{{\hat{\jmath }}}}$, $\mathcal {T}_{\Gamma _{j}}$ and $\mathcal {T}_{\Omega _{{\check{\jmath }}}}$. Grids are formally defined in Sect. 4.1

Motivated by filling this gap, we extend the estimates derived in [20] to the non-matching case. In our context, *non-matching* means that elements in the higher-dimensional grid, the lower-dimensional grid, or both, do not geometrically align with the elements of the coupling interface grid (also known as a mortar grid). However, it is important to remark that all these grids are individually conforming. Figure 1 illustrates this concept in two dimensions. Non-matching grids enable independent refinement or coarsening of subdomains and interfaces, potentially leading to significant computational savings. For instance, one may refine only one subdomain in response to large local estimator values, while leaving the others unchanged. Such fully disjoint adaptivity avoids the technical complexity of enforcing geometric inter-dimensional conformity.

Extending the validity of the *a posteriori* error estimators from the matching to the general non-matching case is a non-trivial task, as one needs to introduce appropriate mappings of the primal (potentials) and dual (fluxes) variables between subdomains and interfaces with different partitioning. In this work, we overcome this challenge by constructing a *transfer grid* (common refinement grid between subdomains and interfaces), from which potentials and interface fluxes are projected in an energy-consistent manner. This approach allows the estimates of [20] to carry over to the non-matching setting modulo the introduction of grid mismatch terms. Optimal generation of transfer grids, however, remains a challenging task [21–23].

The remainder of the article is organized as follows. In Sect. 2, we present the mD geometric decomposition together with the strong formulations. Section 3 introduces the functional framework, including local and mD spaces and continuous transfer operators. Section 4 covers the discrete setting, formally introducing the grids, discrete projection and grid mismatch operators. In Sect. 5, we present the main *a posteriori* error bounds. Section 6 makes the error bounds concrete for primal and dual approximations and discusses potential and flux reconstructions. Finally, in Sect. 7, we present the numerical results and in Sect. 8 we draw our conclusions.

## Mixed-dimensional setting and the model problem

In this section, we introduce the mD decomposition of the domain together with the strong forms of the model problem.

### Mixed-dimensional decomposition

Following the notation introduced in [20], we consider an mD domain $Y\subset \mathbb {R}^n$, $n\in \{2, 3\}$ decomposed into strictly disjoint and flat subdomains $\Omega _i$. Each $\Omega _i$ has a known dimension $d_i = d(i)$, where $0 \le d_i \le n$. There are $N_\Omega$ subdomains and we denote by $I=\{1, 2, \ldots , N_\Omega \}$ the index set of all subdomains. As shown in the left panel of Fig. 1, interfaces $\Gamma _j$ establish the connection between two neighboring subdomains that are one dimension apart. Each interface has a known dimension $d_j = d(j)$, where $0 \le d_j \le n-1$. There are $N_\Gamma$ interfaces and the index set of interfaces is denoted by $J=\{1, 2, \ldots , N_\Gamma \}$. For a given interface $\Gamma _j$, we use the indices ${\hat{\jmath }}\in I$ and ${\check{\jmath }}\in I$ to respectively identify higher- and lower-dimensional neighboring subdomains $\Omega _{{\hat{\jmath }}}$ and $\Omega _{{\check{\jmath }}}$. The portion of the boundary of $\Omega _{{\hat{\jmath }}}$ geometrically coinciding with $\Gamma _j$ is denoted by $\partial _j\Omega _{{\hat{\jmath }}}$. Throughout the article, we assume geometric compatibility of the coupling triplet $(\partial _j\Omega _{{\hat{\jmath }}}, \Gamma _j, \Omega _{{\check{\jmath }}})$ in the following sense.

#### Assumption 1

(Geometric compatibility of the coupling triplet) For each j∈J, the sets

$$ \partial _j\Omega _{\hat{\jmath }},\qquad \Gamma _j,\qquad \Omega _{\check{\jmath }} $$

have the same intrinsic dimension and are pairwise bi-Lipschitz equivalent. In particular, they are regarded as different representations of the same geometric object.

**Fig. 2:**
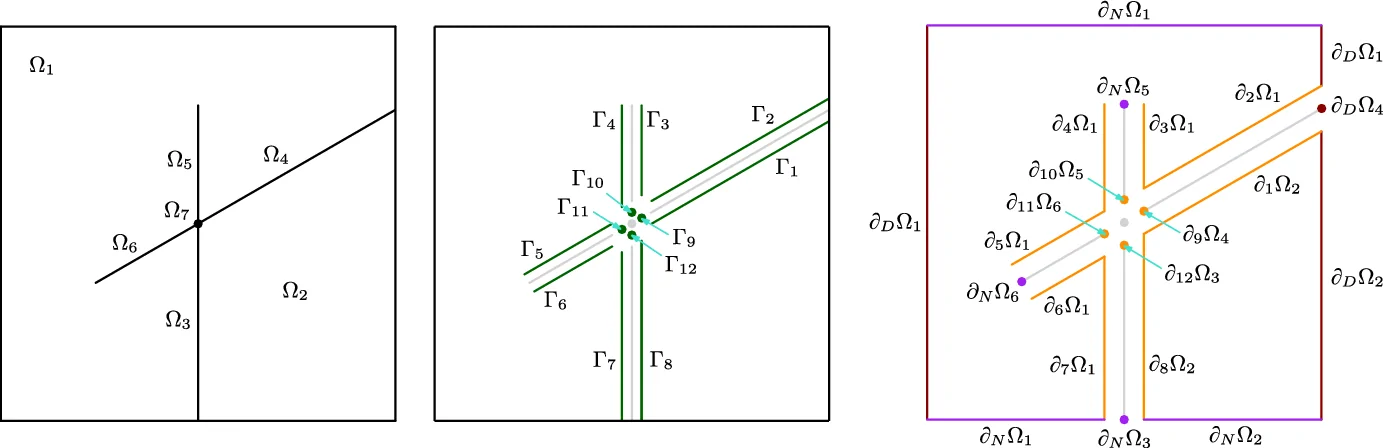
Complete geometric decomposition of an mD domain. Left: Subdomains. Two 2D subdomains ($\Omega _1$ and $\Omega _2$) hosting four 1D fractures ($\Omega _3$, $\Omega _4$, $\Omega _5$, $\Omega _6$) that intersect at a common 0D point ($\Omega _7$). Center: Interfaces. Twelve interfaces coupling neighboring subdomains of co-dimension one. Right: Internal and external boundary conditions

Given that several interfaces can be associated to a single subdomain, for all i∈I, we introduce the interface index sets $\hat{S}_i$ and $\check{S}_i$ to establish their connections. The set $\hat{S}_i$ contains the indices of all $d_i$-dimensional neighboring interfaces of $\Omega _i$, whereas $\check{S}_i$ contains the indices of all $(d_i-1)$-dimensional neighboring interfaces of $\Omega _i$. For example, in Fig. 2, $\hat{S}_5=\{4, 3\}$ and $\check{S}_5=\{10\}$.

Recalling that A⊔B denotes the disjoint union of the sets *A* and *B*, we define the set of all subdomains by $\Omega = \sqcup _{i\in I} \Omega _i$ and the set of all interfaces by $\Gamma = \sqcup _{j\in J}\Gamma _j$. Thus, a complete disjoint partitioning of the mD domain *Y* is given by $Y = \Omega \sqcup \Gamma$.

To finalize the geometric description, we define the boundary of Ω as $\partial \Omega = \partial _D\Omega \cup \partial _N\Omega \cup \partial _\mathcal {I}\Omega$, where $\partial _D\Omega = \cup _{i\in I}\partial _D\Omega _i$, $\partial _N\Omega = \cup _{i\in I}\partial _N\Omega _i$ and $\partial _\mathcal {I}\Omega =\cup _{i\in I} \cup _{j\in \hat{S}_i} \partial _{j}\Omega _i$ are respectively the Dirichlet, Neumann and internal portions of $\partial \Omega$, assumed to be strictly non-overlapping. Since the problem we are interested in is purely elliptic, we require $\partial _D\Omega \ne \emptyset$. A full geometric decomposition of an mD domain $Y\subset \mathbb {R}^2$ is shown schematically in Fig. 2.

### The model problem

We now present the mD elliptic problem in strong form, in the context of incompressible single phase flow in fractured porous media. For the details, we refer to [2, 3, 24].

#### Problem 1

(Strong primal form) Let i∈I and j∈J. Then, find each pressure $p_i: \Omega _i \rightarrow \mathbb {R}$, such that

1a $$\begin{aligned}&-\nabla _i\cdot \left( \mathcal {K}_i\nabla _ip_i\right) + \sum _{j\in \hat{S}_i} \kappa _j\left( p_{{\check{\jmath }}} - p_{{\hat{\jmath }}}\right) = f_i  &   \qquad \textrm{in}~\Omega _i, \end{aligned}$$

1b $$\begin{aligned}&\left( \mathcal {K}_{{\hat{\jmath }}}\nabla _{{\hat{\jmath }}}p_{{\hat{\jmath }}}\right) \cdot {\boldsymbol{n}}_{{\hat{\jmath }}} = \kappa _j \left( p_{{\check{\jmath }}} - p_{{\hat{\jmath }}}\right)  &   \qquad \textrm{on}~\partial _j\Omega _{{\hat{\jmath }}}, \end{aligned}$$

1c $$\begin{aligned}&\left( \mathcal {K}_{i}\nabla _{i}p_{i}\right) \cdot {\boldsymbol{n}}_{i} = 0  &   \qquad \textrm{on}~ \partial _N\Omega _i, \end{aligned}$$

1d $$\begin{aligned}&p_i = g_{D, i}  &   \qquad \textrm{on}~\partial _D\Omega _i. \end{aligned}$$

Introducing the subdomain Darcy (tangential) flux ${\boldsymbol{u}}_i = -\mathcal {K}_i\nabla _ip_i$ and the interface (normal) flux $\lambda _j = -\kappa _j(p_{{\check{\jmath }}} - p_{{\hat{\jmath }}})$, Problem 1 can be recast into the following dual problem.

#### Problem 2

(Strong dual form) Let i∈I and j∈J. Then, find each Darcy flux ${\boldsymbol{u}}_i: \Omega _i \rightarrow \mathbb {R}^{d_i}$, interface flux $\lambda _j: \Gamma _j \rightarrow \mathbb {R}$, and pressure $p_i: \Omega _i \rightarrow \mathbb {R}$, such that

2a $$\begin{aligned}&\nabla _i \cdot {\boldsymbol{u}}_i - \sum _{j \in \hat{S}_i} \lambda _j = f_i  &   \qquad \textrm{in}~\Omega _i, \end{aligned}$$

2b $$\begin{aligned}&{\boldsymbol{u}}_i = -\mathcal {K}_i \nabla _i p_i  &   \qquad \textrm{in}~\Omega _i, \end{aligned}$$

2c $$\begin{aligned}&\lambda _j = -\kappa _j (p_{{\check{\jmath }}} - p_{{\hat{\jmath }}})  &   \qquad \textrm{on}~\Gamma _j, \end{aligned}$$

2d $$\begin{aligned}&{\boldsymbol{u}}_{{\hat{\jmath }}} \cdot {\boldsymbol{n}}_{{\hat{\jmath }}} = \lambda _j  &   \qquad \textrm{on}~\partial _j \Omega _{{\hat{\jmath }}}, \end{aligned}$$

2e $$\begin{aligned}&{\boldsymbol{u}}_i \cdot {\boldsymbol{n}}_i = 0  &   \qquad \textrm{on}~\partial _N \Omega _i, \end{aligned}$$

2f $$\begin{aligned}&p_i = g_{D,i}  &   \qquad \textrm{on}~\partial _D \Omega _i. \end{aligned}$$

In Problems 1 and 2, $\nabla _i$ is the $d_i$-dimensional *nabla* operator, ${\boldsymbol{n}}_i$ the outward-pointing normal vector on a given Neumann boundary $\partial _N\Omega _i$ or internal boundary $\partial _\mathcal {I}\Omega _i$, $f_i: \Omega _i \rightarrow \mathbb {R}$ is a source term, and $g_{D,i}: \partial _D \Omega _i \rightarrow \mathbb {R}$ is the Dirichlet boundary data. Finally, $\mathcal {K}_i: \Omega _i \rightarrow \mathbb {R}^{d_i \times d_i}$ is the effective permeability matrix and $\kappa _j: \Gamma _j \rightarrow \mathbb {R}$ is the effective normal permeability. For the physical interpretation of the model, the reader is referred to [3, 20, 25] and the references therein.

#### Remark 1

The dual form provides a natural framework for coupling non-matching grids, since the introduction of the interface flux $\lambda _j$ naturally decouples subdomain and interface contributions while strongly imposing inter-dimensional flux continuity.

## Functional framework

We now define the functional spaces required to formulate the weak versions of Problems 1 and 2, respectively. From this point onward, we shall use $\nabla _i(\cdot )$ and $\nabla _i\cdot (\cdot )$ to denote the $d_i$-dimensional weak gradient and divergence operators, respectively.

### Local and global spaces

Let *S* denote an open and bounded domain. We use $L^2(S)$ to denote the space of square-integrable functions on *S*, equipped with inner product ${\left( {\cdot },{\cdot }\right) }_S \equiv {\left( {\cdot },{\cdot }\right) }_{L^2(S)}$ and norm $\left\Vert \cdot \right\Vert _S \equiv \left\Vert \cdot \right\Vert _{L^2(S)}$. We also introduce the space of square-integrable vector-valued functions on *S*, that is $\textbf{L}^2(S) = [L^2(S)]^{d(S)}$, with *d*(*S*) denoting the intrinsic dimension of *S*. For the sake of compactness we also use ${\left( {\cdot },{\cdot }\right) }_S \equiv {\left( {\cdot },{\cdot }\right) }_{\textbf{L}^2(S)}$ and $\left\Vert \cdot \right\Vert _S \equiv \left\Vert \cdot \right\Vert _{\textbf{L}^2(S)}$ in the vector case.

The standard energy space is defined as $H^1(\Omega _i)= \{q_i \in L^2(\Omega _i): \nabla _i q_i \in \textbf{L}^2(\Omega _i)\}$ while $H^1_0(\Omega _i)= \{q_i \in H^1(\Omega _i): q_i|_{\partial _D\Omega _i} = 0\}$ denotes the subspace of $H^1(\Omega _i)$ of functions with vanishing traces on Dirichlet boundaries. Additionally, we let $g_i \in H^1(\Omega _i)$ represent any function whose restriction on $\partial _D\Omega _i$ matches the boundary data, i.e., $g_i|_{\partial _D\Omega _i} = g_{D,i}$. Here, $(~\cdot ~)|_{\partial _D\Omega _i}: H^1(\Omega _i)\rightarrow H^{1/2}(\partial _D\Omega _i)$ is the trace operator with $H^{1/2}(\partial _D\Omega _i)$ denoting the fractional Sobolev space on the Dirichlet boundary. We also define the local affine space whose trace on $\partial _D\Omega _i$ satisfies the Dirichlet boundary data as $H^1_0(\Omega _i)+g_i = \{\tilde{w}_i = w_i + g_i: w_i \in H^1_0(\Omega _i)\}$. Although implicit, notice that traces on internal boundaries also belong to the space $H^{1/2}(\partial _{\mathcal {I}}\Omega _i)$.

We now turn our focus to the flux spaces. Define the space $\textbf{H}(\textrm{div},\Omega _i)= \{{\boldsymbol{v}}_i \in \textbf{L}^2(\Omega _i): \nabla _i \cdot {\boldsymbol{v}}_i \in L^2(\Omega _i)\}$ alongside the subspace $\textbf{V}_i= \{{\boldsymbol{v}}_i \in \textbf{H}(\textrm{div},\Omega _i): ({\boldsymbol{v}}_i \cdot {\boldsymbol{n}}_i)|_{\partial _N\Omega _i} = 0\}$, which enforces vanishing traces on Neumann boundaries. Similarly, the space $\textbf{V}_{0,i}= \{{\boldsymbol{v}}_i \in \textbf{V}_i: ({\boldsymbol{v}}_i \cdot {\boldsymbol{n}}_i)|_{\partial _\mathcal {I}\Omega _i} = 0\}$ consists of functions with vanishing traces on both Neumann and internal boundaries. To handle internal boundaries, following [24], we introduce the linear extension operator $\mathcal {R}_j: L^2(\Gamma _j)\rightarrow \textbf{V}_{{\hat{\jmath }}}$, satisfying for any $\nu _j \in L^2(\Gamma _j)$:

3 $$\begin{aligned} (\mathcal {R}_j \nu _j)|_{\partial _j\Omega _{{\hat{\jmath }}}} \cdot {\boldsymbol{n}}_{{\hat{\jmath }}} = {\left\{ \begin{array}{ll} \nu _j &  \text {on } \partial _j\Omega _{{\hat{\jmath }}}, \\ 0 &  \partial \Omega _{\hat{\jmath }}\setminus \partial _j\Omega _{\hat{\jmath }}\end{array}\right. }. \end{aligned}$$

An explicit form for $\mathcal {R}_j$ can be obtained by solving an auxiliary elliptic problem [24]. We shall revisit this operator at the end of Sect. 3.2. The space $\textbf{V}_{0,i}$, together with the linear extension operator $\mathcal {R}_j$, allow us to represent, in a weak sense, subdomain fluxes ${\boldsymbol{v}}_i\in \textbf{V}_i$ with $({\boldsymbol{v}}_i \cdot {\boldsymbol{n}}_i)|_{\partial _j\Omega _{{\hat{\jmath }}}} = \nu _j$ for all $j\in \check{S}_i$, as ${\boldsymbol{v}}_i = {\boldsymbol{v}}_{0,i} + \textstyle {\sum }_{j\in \check{S}_i} \mathcal {R}_j\nu _j$, with ${\boldsymbol{v}}_{0, i} \in \textbf{V}_{0,i}$ and $\nu _j \in L^2(\Gamma _j)$. The above decomposition suggests the following local space for subdomain fluxes

4 $$\begin{aligned} \textbf{X}_{i}:= \textbf{V}_{0,i}\times \prod _{j\in \check{S}_i} \mathcal {R}_j L^2(\Gamma _j). \end{aligned}$$

#### Remark 2

*(*$\textbf{X}_{i}$ *vs.* $\textbf{V}_i$*)* The space $\textbf{X}_{i}$ has higher regularity compared to $\textbf{V}_i$, as this latter does not contain $L^2$ traces on internal boundaries. This enhanced regularity on internal boundaries allows the construction of pure $L^2$-type projections on coupling triplets in the case of weak dual approximations.

Global spaces are now immediate by taking the Cartesian product of local spaces,

5 $$\begin{aligned} {L^2}(\Omega ) := \prod _{i\in I} L^2(\Omega _i), \qquad L^2(\Gamma ) := \prod _{j\in J} L^2(\Gamma _j). \end{aligned}$$

Analogous definitions hold for $\textbf{L}^2(\Omega )$, $H^1(\Omega )$, $H^1_0(\Omega )$, $H^1_0(\Omega ) + g$, $H^{1/2}(\partial _D\Omega )$, $\textbf{V}$, $\textbf{V}_{0}$, and $\textbf{X}$. We will later use these spaces to represent weak solutions to the model problem in the mD partition $\Omega \sqcup \Gamma$.

### Continuous transfer operators

Let j∈J and consider the coupling triplet $(\partial _j\Omega _{{\hat{\jmath }}},\Gamma _j,\Omega _{{\check{\jmath }}})$. Recall that by Assumption 1, the three sets represent the same geometric domain.

#### Definition 1

(Continuous transfer operators) We define the following bounded linear transfer operators:

(i) Transfers between $\partial _j\Omega _{\hat{\jmath }}$ and $\Gamma _j$:
  $$\begin{aligned} \hspace{-6mm}\mathcal P_{{\hat{\jmath }}}^j : H^{1/2}(\partial _j\Omega _{{\hat{\jmath }}}) \longrightarrow H^{1/2}(\Gamma _j), \qquad \mathcal D_{j}^{{\hat{\jmath }}} : L^2(\Gamma _j) \longrightarrow L^2(\partial _j\Omega _{{\hat{\jmath }}}), \end{aligned}$$
  where $\mathcal {P}_{{\hat{\jmath }}}^j$ is an $H^{1/2}$-identification and $\mathcal {D}_j^{{\hat{\jmath }}}$ is an $L^2$-identification.
(ii) Transfers between $\Omega _{\check{\jmath }}$ and $\Gamma _j$:
  $$\begin{aligned} \mathcal P_{{\check{\jmath }}}^j : H^1(\Omega _{{\check{\jmath }}}) \longrightarrow H^{1/2}(\Gamma _j), \qquad ~~ \mathcal D_{j}^{{\check{\jmath }}} : L^2(\Gamma _j) \longrightarrow L^2(\Omega _{{\check{\jmath }}}), \end{aligned}$$
  where $\mathcal P_{{\check{\jmath }}}^j$ is the standard embedding $H^1(\Omega _{{\check{\jmath }}})\hookrightarrow H^{1/2}(\Gamma _j)$ on the same domain and $\mathcal {D}_j^{\check{\jmath }}$ is an $L^2$-identification.

The operators $\mathcal {D}_j^{{\hat{\jmath }}}$ and $\mathcal {D}_j^{{\check{\jmath }}}$ preserve the $L^2$ norm under identification:

$$ \Vert \mathcal D_{j}^{{\hat{\jmath }}} \nu \Vert _{\partial _j\Omega _{{\hat{\jmath }}}}\hspace{-1mm}=\Vert \nu \Vert _{\Gamma _j}, \qquad \Vert \mathcal D_{j}^{{\check{\jmath }}} \nu \Vert _{\Omega _{{\check{\jmath }}}}\hspace{-1mm}=\Vert \nu \Vert _{\Gamma _j}, $$

while the operator $\mathcal P_{{\hat{\jmath }}}^j$ preserves the $H^{1/2}$ norm under identification

$$ \Vert \mathcal P_{{\hat{\jmath }}}^j q\Vert _{H^{1/2}(\Gamma _j)} =\Vert q\Vert _{H^{1/2}\left( \partial _j\Omega _{{\hat{\jmath }}}\right) }. $$

The operator $\mathcal P_{{\check{\jmath }}}^j$ is stable under the Sobolev embedding $H^1(\Omega _{{\check{\jmath }}})\hookrightarrow H^{1/2}(\Gamma _j)$ on the same domain

$$\Vert \mathcal P_{{\check{\jmath }}}^j q\Vert _{H^{1/2}(\Gamma _j)} \le C_{\textrm{emb}}\,\Vert q\Vert _{H^1\left( \Omega _{{\check{\jmath }}}\right) }. $$

Here, $\left\Vert \cdot \right\Vert _{H^1(S)}$ and $\left\Vert \cdot \right\Vert _{H^{1/2}(S)}$ are the usual Sobolev norms on *S* [26], and $C_{\textrm{emb}}$ is the continuity constant of the embedding $H^1(S) \hookrightarrow H^{1/2}(S)$; see, e.g., [27, Ch. 4, Prop. 4.4].

#### Remark 3

*(Continuous transfers vs. discrete projections)* The operators P and D above act as canonical identifications on the common geometry (with norms associated to the target space). They are introduced to keep notation aligned with the discrete non-matching case (where genuine projections $\widetilde{\mathcal {P}}$ and ${\widetilde{\mathcal {D}}}$ are required), and in Sect. 4.3.1, to define the grid mismatch operators.

With the interface to internal-boundary $L^2$-transfer $\mathcal D_{j}^{{\hat{\jmath }}}(\cdot )$ defined, the subdomain extension can be written as the following composition:

6 $$\begin{aligned} \mathcal R_j = \mathcal R_j^* \circ \mathcal D_{j}^{\hat{\jmath }} : L^2(\Gamma _j) \longrightarrow L^2(\partial _j\Omega _{\hat{\jmath }}) \longrightarrow \textbf{V}_{\hat{\jmath }}, \end{aligned}$$

where $\mathcal R_j^*$ is any bounded lifting from the internal boundary into $\textbf{V}_{\hat{\jmath }}$ (cf. [24]).

### Weak forms

Before stating the weak primal and dual forms of Problems 1 and 2, we make the data regularity explicit in the following assumption. Here and in the following, we shall use the notation [·] to denote an ordered collection of subdomain and/or interface variables, depending on context.

#### Assumption 2

*(Data regularity)*

(i) $$f = [f_i]\in L^2(\Omega )$$.
(ii) $$g_D = [g_{D, i}] \in H^{1/2}(\partial _D\Omega )$$.
(iii) For all i∈I, $\mathcal {K}_i$ is bounded, positive definite and satisfies the usual ellipticity condition
  $$\begin{aligned} c_{\mathcal {K}_i}^2 \left|{\boldsymbol{\xi }}\right|^2 \le \mathcal {K}_i{\boldsymbol{\xi }}\cdot {\boldsymbol{\xi }} \le C_{\mathcal {K}_i}^2 \left|{\boldsymbol{\xi }}\right|^2, \qquad \forall {\boldsymbol{\xi }}\in \mathbb {R}^{d_i}. \end{aligned}$$
(iv) For all j∈J, $\kappa _j$ is non-degenerate with bounds
  $$\begin{aligned} 0< c_{\kappa _j} \le \kappa _j \le C_{\kappa _j} < \infty . \end{aligned}$$

Later, we shall use $c_{\mathcal {K}_i}|_{S}$ to denote the restriction of $c_{\mathcal {K}_i}(\textbf{x})$ on the domain *S*.

#### Problem 3

(Weak primal form) Let $p=[p_i]$ and $g = [g_i] \in H^1(\Omega )$. Then, given $f = [f_i]\in L^2(\Omega )$, find $p \in H^1_0(\Omega ) + g$ such that

7 $$\begin{aligned}&\sum _{i\in I} \left( \mathcal {K}_i\nabla _ip_i, \nabla _iq_i\right) _{\Omega _i} + \sum _{j\in J}\left( \kappa _j( \mathcal {P}_{{\check{\jmath }}}^{j}p_{{\check{\jmath }}} - \mathcal {P}_{{\hat{\jmath }}}^{j}p_{{\hat{\jmath }}}|_{\partial _j\Omega _{{\hat{\jmath }}}}), \mathcal {P}_{{\check{\jmath }}}^{j}q_{{\check{\jmath }}} - \mathcal {P}_{{\hat{\jmath }}}^{j}q_{{\hat{\jmath }}}|_{\partial _j\Omega _{{\hat{\jmath }}}}\right) _{\Gamma _j} \nonumber \\&\quad =\sum _{i\in I} \left( f_i, q_i\right) _{\Omega _i} \qquad \forall q=[q_i]\in H^1_0(\Omega ). \end{aligned}$$

#### Remark 4

*(Well-posedness of the primal form)* The bilinear form associated with the primal weak form is symmetric and, provided Assumption 2 holds, coercive on $H^1_0(\Omega )$. Consequently, the problem is well-posed and equivalent to finding the unique minimizer $p \in H^1_0(\Omega ) + g$ of the energy functional:

$$\begin{aligned} \mathcal {J}(q) = \frac{1}{2}\sum _{i\in I} \left\Vert \mathcal {K}_i^{1/2}\nabla _i q_i\right\Vert ^2_{\Omega _i} + \frac{1}{2}\sum _{j\in J} \left\Vert \kappa _j^{1/2} (\mathcal {P}_{{\check{\jmath }}}^{j}q_{{\check{\jmath }}} - \mathcal {P}_{{\hat{\jmath }}}^{j}q_{{\hat{\jmath }}}|_{\partial _j\Omega _{\hat{\jmath }}})\right\Vert ^2_{\Gamma _j} - \sum _{i\in I} (f_i, q_i)_{\Omega _i}. \end{aligned}$$

#### Problem 4

(Dual weak form) Let ${\boldsymbol{u}} = [{\boldsymbol{u}}_{0, i}, \lambda _j]$ and $p = [p_i]$. Then, given $f=[f_i]\in L^2(\Omega )$ and $g_D = [g_{D,i}] \in H^{1/2}(\partial _D\Omega )$, find ${\boldsymbol{u}} \in \textbf{X}$ and $p\in L^2(\Omega )$ such that

8a $$\begin{aligned}&\sum _{i\in I} {\left( {\mathcal {K}^{-1}_i\left( {\boldsymbol{u}}_{0,i} + \textstyle {\sum }_{j\in \check{S}_i}\mathcal {R}^*_j\circ \mathcal {D}_{j}^{{\hat{\jmath }}}\lambda _j\right) },{{\boldsymbol{v}}_{0,i} + \textstyle {\sum }_{j\in \check{S}_i}\mathcal {R}^*_j\circ \mathcal {D}_{j}^{{\hat{\jmath }}}\nu _j}\right) }_{\Omega _i} \nonumber \\&\qquad - \sum _{i\in I} {\left( {p_i},{\nabla _i\cdot \left( {\boldsymbol{v}}_{0,i}+\textstyle {\sum }_{j\in \check{S}_i}\mathcal {R}^*_j\circ \mathcal {D}_{j}^{{\hat{\jmath }}}\nu _j\right) + \textstyle {\sum }_{j\in \hat{S}_i}\mathcal {D}_{j}^{{\check{\jmath }}}\nu _j}\right) }_{\Omega _i} +\sum _{j\in J} {\left( {\kappa _j^{-1}\lambda _j},{\nu _j}\right) }_{\Gamma _j} \nonumber \\&~~= -\sum _{i\in I} {\left( {g_{D,i}},{\left( {\boldsymbol{v}}_{0,i} \cdot {\boldsymbol{n}}_i\right) |_{\partial _D\Omega _{i}}}\right) }_{\partial _D\Omega _i} \qquad \forall {\boldsymbol{v}}=[{\boldsymbol{v}}_{0, i}, \nu _j] \in \textbf{X}. \end{aligned}$$

8b $$\begin{aligned}&\qquad \sum _{i\in I} {\left( {\nabla _i\cdot \left( {\boldsymbol{u}}_{0,i} + \textstyle {\sum }_{j\in \check{S}_i}\mathcal {R}^*_j\circ \mathcal {D}_{j}^{{\hat{\jmath }}}\lambda _j\right) },{q_i}\right) }_{\Omega _i} - \sum _{i\in I} {\left( {\textstyle {\sum }_{j\in \hat{S}_i}\mathcal {D}_{j}^{{\check{\jmath }}}\lambda _j},{q_i}\right) }_{\Omega _i} \nonumber \\&~~=\sum _{i\in I} {\left( {f_i},{q_i}\right) }_{\Omega _i}\qquad \forall q=[q_i]\in L^2(\Omega ). \end{aligned}$$

#### Remark 5

*(Well-posedness of the dual form)* The dual form exhibits a saddle-point structure. Well-posedness can be proven using inf-sup arguments [24], provided the data satisfies Assumption 2.

We will employ mD energy norms to measure the deviation between the exact weak solutions and admissible approximations. More precisely, for an mD potential function $q=[q_i]\in H^1_0(\Omega )$, we define

9 $$\begin{aligned} {\left| \hspace{-1.0625pt}\left| \hspace{-1.0625pt}\left| q \right| \hspace{-1.0625pt}\right| \hspace{-1.0625pt}\right| }^2 = \sum _{i\in I} \left\Vert \mathcal {K}_i^{1/2}\nabla _iq_i\right\Vert _{\Omega _i}^2 + \sum _{j\in J}\left\Vert \kappa _j^{1/2}\left( \mathcal {P}_{{\check{\jmath }}}^{j}q_{{\check{\jmath }}} - \mathcal {P}_{{\hat{\jmath }}}^{j}q_{{\hat{\jmath }}}|_{\partial _j\Omega _{{\hat{\jmath }}}}\right) \right\Vert _{\Gamma _j}^2, \end{aligned}$$

and for an mD flux function ${\boldsymbol{v}}=[{\boldsymbol{v}}_{0,i}, \nu _j] \in \textbf{L}^2(\Omega )\times L^2(\Gamma )$, we define the energy norm

10 $$\begin{aligned} {\left| \hspace{-1.0625pt}\left| \hspace{-1.0625pt}\left| {\boldsymbol{v}} \right| \hspace{-1.0625pt}\right| \hspace{-1.0625pt}\right| }_*^2 = \sum _{i\in I} \left\Vert \mathcal {K}_i^{-1/2}\left( {\boldsymbol{v}}_{0,i}+\textstyle {\sum }_{j\in \check{S}_i}\mathcal {R}_j^*\circ \mathcal {D}_{j}^{{\hat{\jmath }}}\nu _j\right) \right\Vert _{\Omega _i}^2 + \sum _{j\in J} \left\Vert \kappa _j^{-1/2}\nu _j\right\Vert ^2_{\Gamma _j}. \end{aligned}$$

We will use both norms to present the guaranteed upper bounds on the primal (the mD potential) and dual (the mD flux) variables in Sect. 5.

## Discrete setting

In this section, we formally introduce the grids and define the discrete projection and grid mismatch operators. To treat the most general range of dimensionalities, our exposition assumes *Y* such that $\min _{i\in I} d_i = 0$ and $\max _{i\in I} d_i = n = 3$.

### Grid definitions

For all i∈I, we denote by $\mathcal {T}_{\Omega _{i}}$ a simplicial and strictly non-overlapping partition of $\Omega _i$, such that

$$\begin{aligned} \overline{\Omega _i} = \bigcup _{K\in \mathcal {T}_{\Omega _{i}}} K, \qquad \textrm{dim}(K)\in \{0, 1, 2,3\}. \end{aligned}$$

Similarly, for all j∈J, we denote by $\mathcal {T}_{\Gamma _{j}}$ a simplicial and strictly non-overlapping partition of $\Gamma _j$, such that

$$\begin{aligned} \overline{\Gamma _j} = \bigcup _{K\in \mathcal {T}_{\Gamma _{j}}} K, \qquad \textrm{dim}(K)\in \{0, 1, 2\}. \end{aligned}$$

Let *e* denote a face from the set of faces $\mathcal {E}_K$ of an element $K\in \mathcal {T}_{\Omega _{i}}$. For a fixed j∈J, we define the internal boundary grid as

$$\begin{aligned} \mathcal {T}_{\partial _{j}\Omega _{{\hat{\jmath }}}} := \left( \bigcup _{K\in \mathcal {T}_{\Omega _{{\hat{\jmath }}}}} \bigcup _{e\in \mathcal {E}_K}e\right) \bigcap \partial _{j}{\Omega _{{\hat{\jmath }}}}, \qquad \overline{\partial _{j}\Omega _{{\hat{\jmath }}}} = \bigcup _{K\in \mathcal {T}_{\partial _{j}\Omega _{{\hat{\jmath }}}}} K, \qquad \text {dim}(K)\in \{0,1,2\}. \end{aligned}$$

As illustrated in the right panel of Fig. 1, we allow the grid triplet $(\mathcal {T}_{\partial _{j}\Omega _{{\hat{\jmath }}}},\mathcal {T}_{\Gamma _{j}}, \mathcal {T}_{\Omega _{i}})$ to be non-matching. When necessary, we shall use $K_{X}$ to denote an element of any admissible grid $\mathcal {T}_X$.

To ensure the stability of the discrete projection operators for the primal problem that will be constructed in Sect. 4.2.1, we require all partitions of subdomains and interfaces to be shape-regular.

#### Assumption 3

*(Shape-regularity of mixed-dimensional meshes)* There exists ϑ>0 such that every simplex *K* in each mesh $\mathcal {T}_{\Omega _{i}}$, $\mathcal {T}_{\Gamma _{j}}$, $\mathcal {T}_{\partial _{j}\Omega _{{\hat{\jmath }}}}$ satisfies $h_K/\rho _K \le \vartheta$, where $h_K$ is the diameter and $\rho _K$ the inradius of *K*. All meshes are conforming on their respective domains.

Before defining the discrete projection operators, we need to introduce the *transfer grid* between subdomain and interface grids. This grid facilitates the coupling of non-matching grids by resolving the geometric mismatch between them. We focus our exposition on the 3D case (2D interface grid) as the 2D case (1D interface grid) involves fewer geometric complexities.

#### Definition 2

*(Transfer grid)* Let j∈J and $(\mathcal {T}_{\partial _{j}\Omega _{{\hat{\jmath }}}}, \mathcal {T}_{\Gamma _{j}}, \mathcal {T}_{\Omega _{{\check{\jmath }}}})$ be a coupling grid triplet. We construct the transfer grid $\mathcal {T}_{\Gamma _j, X}$ with $X\in \{\partial _j\Omega _{{\hat{\jmath }}}, \Omega _{{\check{\jmath }}}\}$ via a two-step process. First, identify the set of nodes $\mathcal {V}_{\Gamma _j, X} = \mathcal {V}_{\Gamma _j} \bigcup \mathcal {V}_{X}$ of the transfer grid, with $\mathcal {V}_{\Gamma _j}$ and $\mathcal {V}_{X}$ denoting the set of nodes of $\mathcal {T}_{\Gamma _j}$ and $\mathcal {T}_{X}$, respectively. Next, for each pair of elements $K_{\Gamma _j}$ and $K_X$, define

11 $$\begin{aligned} S_{\Gamma _j, X} := K_{\Gamma _j} \bigcap K_{X}. \end{aligned}$$

The above intersections result in one of the following three cases:

(i) *Polygonal domain*. If $S_{\Gamma _j, X}$ is a triangle, then $K_{\Gamma _j, X}:= S_{\Gamma _j, X}$, and we include it directly in $\mathcal {T}_{\Gamma _j, X}$. Otherwise, $S_{\Gamma _j, X}$ is a non-simplex macro-element and it must be triangulated into simplicial elements, see e.g., [28].
(ii) *Line segment*. If $S_{\Gamma _j, X}$ is a line-segment, it represents a shared boundary between two elements and does not contribute as an element to $\mathcal {T}_{\Gamma _j, X}$.
(iii) *Empty intersection*. If $S_{\Gamma _j, X}$ is void, we ignore it.

**Fig. 3:**
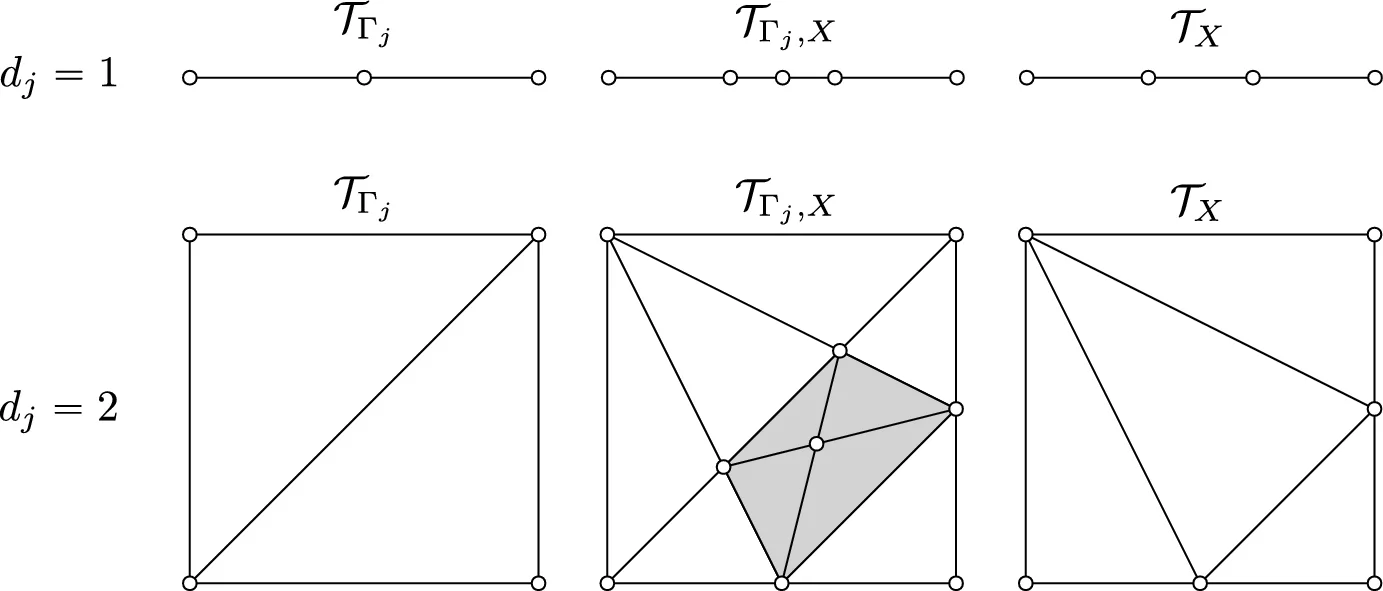
Transfer grids $\mathcal {T}_{\Gamma _j, X}$ coupling interface grids $\mathcal {T}_{\Gamma _j}$ with $\mathcal {T}_{X}$, $X\in \{\partial _j\Omega _{{\hat{\jmath }}},\Omega _{{\check{\jmath }}}\}$. Top: 1D coupling. Bottom: 2D coupling. The shaded quadrilateral corresponds to a macro-element $S_{\Gamma _j, X}$ that was further refined into four simplices using its barycenter coordinate

Figure 3 shows examples of 1D (top panel) and 2D (bottom panel) transfer grids. To finalize this section, we summarize the geometric properties of the transfer grid.

#### Remark 6

*(Properties of the transfer grid)* The transfer grid $\mathcal {T}_{\Gamma _j, X}$ has the properties of node inclusion, refinement, dimensionality, and partitioning:

(i) $$\mathcal {V}_{\Gamma _j} \subseteq \mathcal {V}_{\Gamma _j, X}$$, $$\mathcal {V}_X\subseteq \mathcal {V}_{\Gamma _j, X}$$.
(ii) $$\left|\mathcal {T}_{\Gamma _{j}}\right|\le \left|\mathcal {T}_{\Gamma _j, X}\right|$$, $$\left|\mathcal {T}_{X}\right|\le \left|\mathcal {T}_{\Gamma _j, X}\right|$$.
(iii) For all $K\in \mathcal {T}_{\Gamma _j, X}, ~ \dim (K)=d_j$.
(iv) By Assumption 1, $\overline{\Gamma _j} = \bigcup _{K\in \mathcal {T}_{\Gamma _j, X}} K$, $\overline{X} = \bigcup _{K\in \mathcal {T}_{\Gamma _j, X}} K$.

In Remark 6 (ii), |·| denotes the cardinality of the mesh, i.e., the number of simplicial elements *K*.

### Discrete projection operators

To define the discrete projection operators, we shall make extensive use of the broken spaces of polynomials. For k≥0 and any mesh $\mathcal {T}_X$ on a geometric set *X*, we define

12 $$\begin{aligned} \mathbb {P}_k(\mathcal {T}_X) := \{\, q_h \in L^2(X) \;:\; q_h|_{K} \in \mathbb {P}_k(K)\ \ \forall \, K\in \mathcal {T}_X \,\}. \end{aligned}$$

Functions in $\mathbb {P}_k(\mathcal {T}_X)$ are generally discontinuous across inter-element boundaries and are not required to satisfy any external boundary condition.

#### Discrete projections for the primal problem

The projection of potentials for the weak primal problem on non-matching grids is carried out in two stages. First, potentials defined on the source grids (the internal boundary grids and lower-dimensional subdomain grids) are prolonged onto the corresponding transfer grids. Second, the potentials are projected onto the target interface grid using the Scott-Zhang quasi-interpolant [29].

To be precise, let j∈J be fixed and let $(\partial _{j}\Omega _{{\hat{\jmath }}}, \Gamma _j, \Omega _{{\check{\jmath }}})$ be a coupling triplet satisfying Assumption 1 and let Assumption 3 also be satisfied. For k≥1, we define the prolongation operators

13a $$\begin{aligned} \widetilde{\mathcal {P}}_{{\hat{\jmath }}}^{j,{\hat{\jmath }}}&: \mathbb {P}_k(\mathcal {T}_{\partial _j\Omega _{{\hat{\jmath }}}})\cap H^{1/2}(\partial _j\Omega _{{\hat{\jmath }}}) \longrightarrow \mathbb {P}_k(\mathcal {T}_{\Gamma _j, \partial _j\Omega _{{\hat{\jmath }}}}) \cap H^{1/2}(\partial _j\Omega _{{\hat{\jmath }}}), \end{aligned}$$

13b $$\begin{aligned} \widetilde{\mathcal {P}}_{{\check{\jmath }}}^{j,{\check{\jmath }}}&: \mathbb {P}_k(\mathcal {T}_{\Omega _{{\check{\jmath }}}}) \cap (H^1(\Omega _{{\check{\jmath }}})+g_{{\check{\jmath }}}) \longrightarrow \mathbb {P}_k(\mathcal {T}_{\Gamma _j, \Omega _{{\check{\jmath }}}}) \cap (H^1(\Omega _{{\check{\jmath }}})+g_{{\check{\jmath }}}), \end{aligned}$$

where the transfer grids $\mathcal {T}_{\Gamma _j, \partial _j\Omega _{{\hat{\jmath }}}}$ and $\mathcal {T}_{\Gamma _j, \Omega _{{\check{\jmath }}}}$ are defined as in Definition 2. Two remarks are in order. First, the intersection of a broken polynomial space $\mathbb {P}_k$ with an $H^{1/2}$-space selects the conforming $C^0$ finite element subspace, since jump discontinuities across inter-element boundaries are not admissible in $H^{1/2}$. Second, whenever $\Omega _{\check{\jmath }}$ intersects the external Dirichlet boundary, we assume that the prescribed boundary datum $g_{\check{\jmath }}$ is exactly representable in the corresponding conforming polynomial space. Under this assumption, the affine spaces in (13b) are nonempty and the boundary condition is imposed exactly. The case in which the discrete approximation does not satisfy the Dirichlet boundary condition exactly is outside the scope of the present work; for related discussion in the context of functional a posteriori estimates, see [30, Sec. 4].

Since $\mathcal {T}_{\Gamma _j, X}$ is a subdivision of $\mathcal {T}_{X}$, for any potential $q_h \in \mathbb {P}_k(\mathcal {T}_{\partial _j\Omega _{{\hat{\jmath }}}})\cap H^{1/2}(\partial _j\Omega _{{\hat{\jmath }}})$, (13a) satisfies

14 $$\begin{aligned} (\widetilde{\mathcal {P}}_{{\hat{\jmath }}}^{j,{\hat{\jmath }}}q_h)|_{K_{\Gamma _j, \partial _j\Omega _{{\hat{\jmath }}}}} = q_h |_{K_{\partial _j\Omega _{{\hat{\jmath }}}}}, \qquad \forall K_{\Gamma _j, \partial _{j}\Omega _{{\hat{\jmath }}}} \in S_{\Gamma _{j}, \partial _j\Omega _{\hat{\jmath }}}, \end{aligned}$$

for each transfer macro-element $S_{\Gamma _{j}, \partial _j\Omega _{\hat{\jmath }}}$ and for each $K_{\partial _{j}\Omega _{{\hat{\jmath }}}} \subseteq S_{\Gamma _{j}, \partial _j\Omega _{\hat{\jmath }}}$. The operator $\widetilde{\mathcal {P}}_{{\check{\jmath }}}^{j,{\check{\jmath }}}$ from (13b) satisfies an analogous property for potentials defined on $\mathcal {T}_{\Omega _{{\check{\jmath }}}}$.

Once functions have been prolonged to the transfer grids, we project them onto the target interface grid:

15a $$\begin{aligned} \widetilde{\mathcal {P}}_{j, {\hat{\jmath }}}^{j}&: \mathbb {P}_k(\mathcal {T}_{\Gamma _j, \partial _j\Omega _{{\hat{\jmath }}}}) \cap H^{1/2}(\partial _j\Omega _{{\hat{\jmath }}}) \longrightarrow \mathbb {P}_k(\mathcal {T}_{\Gamma _j})\cap H^{1/2}(\Gamma _{j}), \end{aligned}$$

15b $$\begin{aligned} \widetilde{\mathcal {P}}_{j, {\check{\jmath }}}^{j}&: \mathbb {P}_k(\mathcal {T}_{\Gamma _j, \Omega _{{\check{\jmath }}}}) \cap (H^{1}(\Omega _{{\check{\jmath }}}) +g_{\check{\jmath }}) \longrightarrow \mathbb {P}_k(\mathcal {T}_{\Gamma _j})\cap H^{1/2}(\Gamma _{j}) . \end{aligned}$$

Here, $\widetilde{\mathcal {P}}_{j, {\hat{\jmath }}}^{j}$ and $\widetilde{\mathcal {P}}_{j, {\check{\jmath }}}^{j}$ denote Scott-Zhang quasi-interpolators constructed on $\mathcal {T}_{\Gamma _j}$, see [29] for the details.

##### Definition 3

*(Discrete projections for the primal problem)* Let j∈J and consider the coupling triplet $(\partial _{j}\Omega _{{\hat{\jmath }}}, \Gamma _j, \Omega _{{\check{\jmath }}})$. For a fixed degree k≥1, the discrete projections for the weak primal problem are given by the following compositions

16a $$\begin{aligned} \widetilde{\mathcal {P}}_{{\hat{\jmath }}}^{j}&:= \widetilde{\mathcal {P}}_{j, {\hat{\jmath }}}^{j}\circ \widetilde{\mathcal {P}}_{{\hat{\jmath }}}^{j,{\hat{\jmath }}}, \end{aligned}$$

16b $$\begin{aligned} \widetilde{\mathcal {P}}_{{\check{\jmath }}}^{j}&:= \widetilde{\mathcal {P}}_{j, {\check{\jmath }}}^{j}\circ \widetilde{\mathcal {P}}_{{\check{\jmath }}}^{j,{\check{\jmath }}}. \end{aligned}$$

The following lemma establishes a key stability result for the projection operators.

##### Lemma 1

(Stability of discrete projection operators for the weak primal problem) Let j∈J and k≥1. Then, the operators $\widetilde{\mathcal {P}}_{{\hat{\jmath }}}^j$ and $\widetilde{\mathcal {P}}_{{\check{\jmath }}}^j$ in (16b) are linear and bounded:

$$ \Vert \widetilde{\mathcal {P}}_{{\hat{\jmath }}}^{j}q_h\Vert _{H^{1/2}(\Gamma _j)} \le C_1\, \Vert q_h\Vert _{H^{1/2}(\partial _j\Omega _{{\hat{\jmath }}})}, \qquad \Vert \widetilde{\mathcal {P}}_{{\check{\jmath }}}^{j}\phi _h\Vert _{H^{1/2}(\Gamma _j)} \le C_2\, \Vert \phi _h\Vert _{H^{1}(\Omega _{{\check{\jmath }}})}, $$

with constants $C_1$ and $C_2$ depending only on the shape-regularity of the target interface grid and on the polynomial degree *k*.

##### Proof

The prolongation operators are isometric identifications on an equal or more refined partition of the same domain; thus norm preserving in $H^s$, s∈[0,1] (see e.g., [31, 32]). The Scott-Zhang quasi-interpolant on the target interface grid is linear and $H^s$-stable for s∈[0,1] [29]. Setting s=1/2 yields the first bound. For the second bound, use the Scott-Zhang stability with s=1 and then the continuous embedding $H^1(\Gamma _j) \hookrightarrow H^{1/2}(\Gamma _j)$, cf. Section 4.8 of [33]. □

We emphasize that the stability results in Lemma 1 (and for the ones from the next section) are independent from the transfer grids, as they act purely as data identifiers (stability constants equal to one). This result is numerically verified in Sect. 7.1.

#### Discrete projections for the dual problem

We construct discrete projections for interface fluxes onto the internal boundary $\mathcal {T}_{\partial _j\Omega _{{\hat{\jmath }}}}$ and the lower-dimensional grid $\mathcal {T}_{\Omega _{{\check{\jmath }}}}$, while preserving the elementwise mass on the target grids. The construction consists of (i) a prolongation to a transfer grid and (ii) an agglomeration on the target grid via a constrained $L^2$ projection enforcing cell mean.

Fix j∈J and the coupling triplet $(\partial _j\Omega _{{\hat{\jmath }}}, \Gamma _j, \Omega _{{\check{\jmath }}})$ with transfer grids $\mathcal {T}_{\Gamma _j,\partial _j\Omega _{{\hat{\jmath }}}}$ and $\mathcal {T}_{\Gamma _j,\Omega _{{\check{\jmath }}}}$ from Definition 2. For k≥0 and $\nu _h\in \mathbb {P}_k(\mathcal {T}_{\Gamma _j})$, define the elementwise prolongations

17a $$\begin{aligned} \widetilde{\mathcal {D}}_{j}^{j, {\hat{\jmath }}}&: \mathbb {P}_k(\mathcal {T}_{\Gamma _j}) \longrightarrow \mathbb {P}_k(\mathcal {T}_{\Gamma _j,\partial _j\Omega _{{\hat{\jmath }}}}), \end{aligned}$$

17b $$\begin{aligned} \widetilde{\mathcal {D}}_{j}^{j, {\check{\jmath }}}&: \mathbb {P}_k(\mathcal {T}_{\Gamma _j}) \longrightarrow \mathbb {P}_k(\mathcal {T}_{\Gamma _j,\Omega _{{\check{\jmath }}}}), \end{aligned}$$

by elementwise value restriction: $(\,\cdot \,)|_{S_{\Gamma _j,X}} = (\,\cdot \,)|_{K_{\Gamma _j}}$ for each macro-element $S_{\Gamma _j,X}\subset K_{\Gamma _j}$ and $X\in \{\partial _j\Omega _{{\hat{\jmath }}},\Omega _{{\check{\jmath }}}\}$. Note that the functions prolonged to the transfer grid are in general discontinuous across transfer elements.

Given the prolonged data $\nu _h$ on $\mathcal {T}_{\Gamma _{j}, X}$ for $X \in \{\partial _j\Omega _{\hat{\jmath }}, \Omega _{\check{\jmath }}\}$, we perform a constrained $L^2$ agglomeration on the target cell $K\in \mathcal {T}_{X}$. Let $\{\phi _i^K\}_{i=1}^{N_k}$ be a basis of $\mathbb {P}_k(K)$. Define the local system $M^K\alpha ^K=b^K$, where

18 $$\begin{aligned} M_{ij}^K&= (\phi _i^K, \phi _j^K)_K, \qquad b_i^K  &   = (\nu _h, \phi _i^K)_K = \textstyle {\sum }_{K_{\Gamma _j, X}\subset K} (\nu _h, \phi _i^K)_{K_{\Gamma _j}, X} \end{aligned}$$

19 $$\begin{aligned} c_i^K&=(1, \phi _i^K)_K, \qquad m^K  &   = \textstyle {\sum }_{K_{\Gamma _j, X}\subset K} (\nu _h, 1), \end{aligned}$$

with $M^K$ denoting the local mass matrix, $b^K$ the vector of constants, and $c^K$ and $m^K$ the mass constraints. The coefficients $\alpha ^K$ and the Lagrange multiplier $\mu ^K$ are determined from the local constrained optimization problem

20 $$\begin{aligned} \begin{bmatrix} M^K &  c^K\\ (c^K)^{\!\top } &  0 \end{bmatrix} \begin{bmatrix} \alpha ^K\\ \mu ^K \end{bmatrix} = \begin{bmatrix} b^K \\ m^K \end{bmatrix}. \end{aligned}$$

The unique solvability of this system is guaranteed for any k≥0. The local mass matrix $M^K$ is symmetric positive definite and since $1\in \mathbb {P}_k(K)$, the constraint vector $c^K$ is non-zero. This ensures the satisfaction of the inf-sup condition for the Lagrange multiplier $\mu ^K$. This local constrained optimization problem defines the operators

21a $$\begin{aligned} \widetilde{\mathcal {D}}_{j,{\hat{\jmath }}}^{{\hat{\jmath }}}&: \mathbb {P}_k(\mathcal {T}_{\Gamma _j,\partial _j\Omega _{{\hat{\jmath }}}}) \longrightarrow \mathbb {P}_k(\mathcal {T}_{\partial _j\Omega _{{\hat{\jmath }}}}), \end{aligned}$$

21b $$\begin{aligned} \widetilde{\mathcal {D}}_{j,{\check{\jmath }}}^{{\check{\jmath }}}&: \mathbb {P}_k(\mathcal {T}_{\Gamma _j,\Omega _{{\check{\jmath }}}}) \longrightarrow \mathbb {P}_k(\mathcal {T}_{\Omega _{{\check{\jmath }}}}), \end{aligned}$$

where, on each target element *K*, the image is the unique polynomial in $\mathbb {P}_k(K)$ determined by the constrained local problem (20). Consequently, the resulting polynomial on *K* is $(\widetilde{\mathcal {D}}_{j,{\hat{\jmath }}}^{{\hat{\jmath }}}\nu _h)|_K ~=~ \sum _{i=1}^{N_k} \alpha _i^K \phi _i^K$ and analogously for $\widetilde{\mathcal {D}}_{j,{\check{\jmath }}}^{{\check{\jmath }}}(\cdot )$ on $\mathcal {T}_{\Omega _{\check{\jmath }}}$.

##### Definition 4

*(Discrete projections of interface fluxes for the dual problem)* Let j∈J and consider the coupling triplet $(\partial _{j}\Omega _{{\hat{\jmath }}}, \Gamma _j, \Omega _{{\check{\jmath }}})$. For a fixed degree k≥0, the discrete projections of interface fluxes for the weak dual problem are given by compositions

22a $$\begin{aligned} \widetilde{\mathcal {D}}_{j}^{{\hat{\jmath }}}&:=~ \widetilde{\mathcal {D}}_{j,{\hat{\jmath }}}^{{\hat{\jmath }}}\circ \widetilde{\mathcal {D}}_{j}^{j, {\hat{\jmath }}}, \end{aligned}$$

22b $$\begin{aligned} \widetilde{\mathcal {D}}_{j}^{{\check{\jmath }}}&:=~ \widetilde{\mathcal {D}}_{j,{\check{\jmath }}}^{{\check{\jmath }}}\circ \widetilde{\mathcal {D}}_{j}^{j, {\check{\jmath }}}. \end{aligned}$$

The resulting mapping preserves elementwise mass and inherits stability from the constrained $L^2$ projection.

##### Lemma 2

(Stability of discrete projections of interface fluxes for the dual problem) The operators $\widetilde{\mathcal {D}}_{j}^{{\hat{\jmath }}}$ and $\widetilde{\mathcal {D}}_{j}^{{\check{\jmath }}}$ are linear, $L^2$–stable, and satisfy

23 $$\begin{aligned} \Vert \widetilde{\mathcal {D}}_{j}^{{\hat{\jmath }}}\nu _h\Vert _{\partial _j\Omega _{{\hat{\jmath }}}} \le C_3\, \Vert \nu _h\Vert _{\Gamma _j}, \qquad \Vert \widetilde{\mathcal {D}}_{j}^{{\check{\jmath }}}\nu _h\Vert _{\Omega _{\check{\jmath }}} \le C_4\, \Vert \nu _h\Vert _{\Gamma _j}, \end{aligned}$$

with positive constants $C_3$ and $C_4$. Moreover, for each $K\in \mathcal {T}_{\partial _j\Omega _{\hat{\jmath }}}$, the local polynomial $(\widetilde{\mathcal {D}}_{j}^{{\hat{\jmath }}}\nu _h)|_K$ is characterized as the unique solution of the constrained minimization problem

24 $$\begin{aligned} (\widetilde{\mathcal {D}}_{j}^{{\hat{\jmath }}}\nu _h)|_K = \arg \min _{\varphi \in \mathbb {P}_k(K)} \bigl \{\Vert \varphi - \nu _h\Vert _{K} : {\left( {\varphi },{1}\right) }_K = m^K \bigr \}, \end{aligned}$$

and an analogous statement holds for $\widetilde{\mathcal {D}}_{j}^{{\check{\jmath }}}(\cdot )$ on $\mathcal {T}_{\Omega _{\check{\jmath }}}$.

##### Proof

The prolongation step acts as an isometric identification on each transfer sub-element and is therefore $L^2$-stable. The agglomeration onto the target grid is a constrained $L^2$–orthogonal projection, hence locally stable and the best-approximation property holds by orthogonality. Global bounds follow from the fact that, for each target element $K \in \mathcal {T}_{X}$, $X\in \{\partial _j\Omega _{{\hat{\jmath }}}, \Omega _{\check{\jmath }}\}$, only finitely many transfer elements $K_{\Gamma _j, X} \subset K$ contribute to the local projection. □

Importantly, the discrete projection operator $\widetilde{\mathcal {D}}_{j}^{{\hat{\jmath }}}$ is locally mass-conservative, in the sense that for any $\nu _h \in \mathbb {P}_k(\mathcal {T}_{\Gamma _j})$, there holds

25 $$\begin{aligned} (\widetilde{\mathcal {D}}_{j}^{{\hat{\jmath }}}\nu _h, 1)_K = m^K \quad \text {with}\quad m^K = \textstyle {\sum }_{K_{\Gamma _j, X} \subset K} ~ {\left( {\nu _h},{1}\right) }_{K_{\Gamma _j, X}}. \end{aligned}$$

That is, the total interface flux over *K* equals the sum of incoming fluxes on all transfer grid elements $K_{\Gamma _j, \partial _j\Omega _{\hat{\jmath }}}$ contained in *K*. The same property holds for $\widetilde{\mathcal {D}}_{j}^{{\check{\jmath }}}(\cdot )$ on $\mathcal {T}_{\Omega _{\check{\jmath }}}$.

##### Remark 7

*(Discrete projections of interface fluxes for dual approximations of the lowest-order)* In the lowest-order case k=0, (20) reduces to the overlap-weighted average on each target element:

$$\begin{aligned} (\widetilde{\mathcal {D}}_{j,{\hat{\jmath }}}^{{\hat{\jmath }}}\nu _h)|_K = \frac{1}{|K|} \sum _{K_{\Gamma _j, \partial _j\Omega _{\hat{\jmath }}} \subset K} {\left( {\nu _h},{1}\right) }_{K_{\Gamma _j, \partial _j\Omega _{\hat{\jmath }}}} = \frac{1}{|K|} \sum _{K_{\Gamma _j, \partial _j\Omega _{\hat{\jmath }}} \subset K} |K_{\Gamma _j, \partial _j\Omega _{\hat{\jmath }}}|~ \nu _h|_{K_{\Gamma _j, \partial _j\Omega _{\hat{\jmath }}}}. \end{aligned}$$

An analogous property holds for projection onto the low-dimensional subdomain grid.

**Fig. 4:**
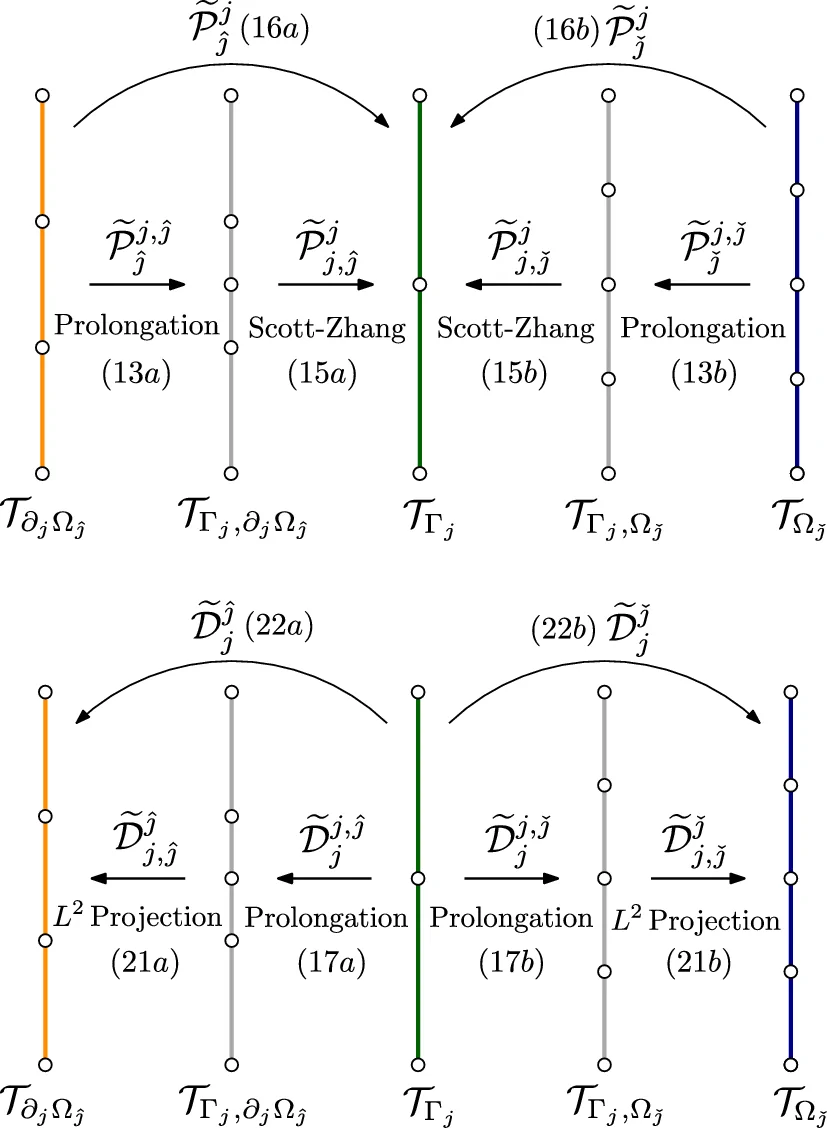
Schematic representation of the discrete projection operators for the weak primal and dual problems. In each case, the construction is decomposed into a prolongation to a transfer grid followed by a projection onto the target grid. Curved arrows indicate the corresponding composite discrete operators. Equation numbers refer to the formal definitions in the text

Figure 4 shows the four discrete projection operators introduced in this section.

### Grid mismatch operators

This subsection introduces the grid mismatch (GM) operators required to evaluate the local error indicators in Sect. 5. Each GM operator quantifies the discrepancy between a continuous transfer operator from Definition 1 and its discrete realization from Sects. 4.2.1–4.2.2 on a non-matching coupling triplet $(\partial _j\Omega _{{\hat{\jmath }}}, \Gamma _j, \Omega _{{\check{\jmath }}})$. We also fix the convention by which the discrete lifting acts on grid-mismatch data, and prove a cellwise mass-defect property essential for the proof of Theorem 1.

#### Defect operators

Let j∈J and consider the coupling triplet $(\partial _j\Omega _{{\hat{\jmath }}}, \Gamma _j, \Omega _{{\check{\jmath }}})$. The defect operators are constructed on the transfer grids of Definition 2 as the difference between a continuous transfer operator and its discrete realization. Before stating the construction, we recall a key structural property of the transfer grids.

##### Remark 8

*(Transfer grids as common refinements)* By Remark 6, the transfer grids satisfy $\mathcal {V}_{\Gamma _j} \cup \mathcal {V}_{\partial _j\Omega _{{\hat{\jmath }}}} \subseteq \mathcal {V}_{\Gamma _j,\partial _j\Omega _{{\hat{\jmath }}}}$ and $\mathcal {V}_{\Gamma _j} \cup \mathcal {V}_{\Omega _{{\check{\jmath }}}} \subseteq \mathcal {V}_{\Gamma _j,\Omega _{{\check{\jmath }}}}$, that is, $\mathcal {T}_{\Gamma _j,\partial _j\Omega _{{\hat{\jmath }}}}$ and $\mathcal {T}_{\Gamma _j,\Omega _{{\check{\jmath }}}}$ are the *common refinements* of the interface grid $\mathcal {T}_{\Gamma _j}$ with the internal-boundary grid $\mathcal {T}_{\partial _j\Omega _{{\hat{\jmath }}}}$ and with the lower-dimensional subdomain grid $\mathcal {T}_{\Omega _{{\check{\jmath }}}}$, respectively. Consequently, any piecewise polynomial on $\mathcal {T}_{\Gamma _j}$ admits a pointwise-equal representation as a piecewise polynomial on $\mathcal {T}_{\Gamma _j,\partial _j\Omega _{{\hat{\jmath }}}}$ (and on $\mathcal {T}_{\Gamma _j,\Omega _{{\check{\jmath }}}}$), and the same holds for any piecewise polynomial on $\mathcal {T}_{\partial _j\Omega _{{\hat{\jmath }}}}$ or $\mathcal {T}_{\Omega _{{\check{\jmath }}}}$. The transfer grids are therefore the natural common partitions on which the continuous identifications $\mathcal {D},\mathcal {P}$ and the discrete realizations $\widetilde{\mathcal {D}},\widetilde{\mathcal {P}}$ admit a simultaneous, pointwise-well-defined representation, so that their pointwise difference is unambiguously a piecewise polynomial on the corresponding transfer grid.

##### Definition 5

*(Grid mismatch operators)* For $q_{\hat{\jmath }}\in \mathbb {P}_k(\mathcal {T}_{\partial _j\Omega _{\hat{\jmath }}}) \cap H^{1/2}(\partial _j\Omega _{\hat{\jmath }})$, $q_{\check{\jmath }}\in \mathbb {P}_k(\Omega _{\check{\jmath }}) \cap (H^{1}(\Omega _{\check{\jmath }}) + g_{\check{\jmath }})$ and $\nu _j \in \mathbb {P}_k(\mathcal {T}_{\Gamma _j})$, the primal and dual grid-mismatch operators associated with the coupling triplet $(\partial _j\Omega _{{\hat{\jmath }}}, \Gamma _j, \Omega _{{\check{\jmath }}})$ are defined elementwise on the transfer grids by

26a $$\begin{aligned} \delta \mathcal {P}^j_{{\hat{\jmath }}}q_{{\hat{\jmath }}}&:= (\mathcal {P}^j_{{\hat{\jmath }}}q_{{\hat{\jmath }}} - \widetilde{\mathcal {P}}^j_{{\hat{\jmath }}}q_{{\hat{\jmath }}}) \in \mathbb {P}_{k}(\mathcal {T}_{\Gamma _j,\partial _j\Omega _{{\hat{\jmath }}}}) \cap H^{1/2}(\mathcal {T}_{\Gamma _j,\partial _j\Omega _{{\hat{\jmath }}}}), \quad  &   k\ge 1,\end{aligned}$$

26b $$\begin{aligned} \delta \mathcal {P}^j_{{\check{\jmath }}}q_{{\check{\jmath }}}&:= (\mathcal {P}^j_{{\check{\jmath }}}q_{{\check{\jmath }}} - \widetilde{\mathcal {P}}^j_{{\check{\jmath }}}q_{{\check{\jmath }}}) \in \mathbb {P}_k(\mathcal {T}_{\Gamma _j,\Omega _{{\check{\jmath }}}}) \cap H^{1/2}(\mathcal {T}_{\Gamma _j,\Omega _{{\check{\jmath }}}}),\quad  &   k\ge 1, \end{aligned}$$

26c $$\begin{aligned} \delta \mathcal {D}_j^{{\hat{\jmath }}}\nu _j&:= (\mathcal {D}_j^{{\hat{\jmath }}}\nu _j - \widetilde{\mathcal {D}}_j^{{\hat{\jmath }}}\nu _j) \in \mathbb {P}_k(\mathcal {T}_{\Gamma _j,\partial _j\Omega _{{\hat{\jmath }}}}), \quad  &   k \ge 0, \end{aligned}$$

26d $$\begin{aligned} \delta \mathcal {D}_j^{{\check{\jmath }}}\nu _j&:= (\mathcal {D}_j^{{\check{\jmath }}}\nu _j - \widetilde{\mathcal {D}}_j^{{\check{\jmath }}}\nu _j) \in \mathbb {P}_k(\mathcal {T}_{\Gamma _j,\Omega _{{\check{\jmath }}}}), \quad  &   k\ge 0, \end{aligned}$$

where, in each formula, both terms on the right-hand side are evaluated on the transfer grid in the sense of Remark 8: the continuous identification $\mathcal {P}^j_\bullet q_i$ (resp. $\mathcal {D}_j^\bullet \nu _j$ ) is the piecewise polynomial on the source grid prolonged elementwise to the transfer grid, and the discrete realization $\widetilde{\mathcal {P}}^j_\bullet q_i$ (resp. $\widetilde{\mathcal {D}}_j^\bullet \nu _j$) is the piecewise polynomial on the target grid prolonged back elementwise to the transfer grid.

By Remark 8, both prolongations to the transfer grid are isometric identifications: they preserve the $L^2$ (resp. $H^{1/2}$) norm and introduce no information loss. The defect operators in Definition 5 are therefore well-defined pointwise on their respective transfer grids and vanish identically in the matching case $\mathcal {T}_{\partial _j\Omega _{{\hat{\jmath }}}} = \mathcal {T}_{\Gamma _j} = \mathcal {T}_{\Omega _{{\check{\jmath }}}}$ (see also Corollary 1). Their stability follows from the triangle inequality applied to the underlying continuous identifications (cf. Definition 1) and discrete projections (cf. Lemmas 1 and 2).

#### Lifting of grid mismatch data

The local GM error indicators that will be presented in Theorem 1 contain the bulk lift $\widetilde{\mathcal {R}}^*_j\circ \delta \mathcal {D}_j^{{\hat{\jmath }}}\nu _j$. The discrete lift $\widetilde{\mathcal {R}}^*_j$ is, by construction in [24], defined on functions in $\mathbb {P}_k(\mathcal {T}_{\partial _j\Omega _{{\hat{\jmath }}}})$, whereas $\delta \mathcal {D}_j^{{\hat{\jmath }}}\nu _j$, by (26c), can in general only be resolved on the finer transfer grid $\mathcal {T}_{\Gamma _j,\partial _j\Omega _{{\hat{\jmath }}}}$. To resolve this domain mismatch, we abuse notation and adopt the convention that $\widetilde{\mathcal {R}}^*_j$ acts on transfer-grid data by precomposition with the constrained $L^2$ projection $\widetilde{\mathcal {D}}_{j,{\hat{\jmath }}}^{{\hat{\jmath }}}$ from Sect. 4.2.2.

##### Definition 6

*(Lifting of grid mismatch data)* For $w\in \mathbb {P}_k(\mathcal {T}_{\Gamma _j,\partial _j\Omega _{{\hat{\jmath }}}})$, the lift of *w* into the bulk subdomain $\Omega _{{\hat{\jmath }}}$ is defined as

27 $$\begin{aligned} \widetilde{\mathcal {R}}^*_j(w) \;:=\; \widetilde{\mathcal {R}}^*_j\bigl (\widetilde{\mathcal {D}}_{j,{\hat{\jmath }}}^{{\hat{\jmath }}}\, w\bigr ) \;\in \; \textbf{V}_{h, {\hat{\jmath }}}, \end{aligned}$$

where $\textbf{V}_{h, {\hat{\jmath }}}$ is a suitable discrete subspace of $\textbf{V}_{\hat{\jmath }}$.

The convention (27) is well-posed because $\widetilde{\mathcal {D}}_{j,{\hat{\jmath }}}^{{\hat{\jmath }}}$ is the orthogonal $L^2$ projection from a refinement of $\mathcal {T}_{\partial _j\Omega _{{\hat{\jmath }}}}$ onto $\mathcal {T}_{\partial _j\Omega _{{\hat{\jmath }}}}$ itself, and is therefore $L^2$-stable with constant equal to one,

28 $$\begin{aligned} \Vert \widetilde{\mathcal {D}}_{j,{\hat{\jmath }}}^{{\hat{\jmath }}}\, w\Vert _{\partial _j\Omega _{{\hat{\jmath }}}} \;\le \; \Vert w\Vert _{\partial _j\Omega _{{\hat{\jmath }}}}, \qquad w\in \mathbb {P}_k(\mathcal {T}_{\Gamma _j,\partial _j\Omega _{{\hat{\jmath }}}}). \end{aligned}$$

Composition with $\widetilde{\mathcal {R}}^*_j$ therefore introduces no nonexplicit constant: the lift (27) inherits the computability of $\widetilde{\mathcal {R}}^*_j$ on its native domain $\mathbb {P}_k(\mathcal {T}_{\partial _j\Omega _{{\hat{\jmath }}}})$.

We further require the discrete lift to be *locally compatible* with the source-boundary data, in the following sense.

##### Assumption 4

*(Cell-balance of the discrete lift)* For every $w\in \mathbb {P}_k(\mathcal {T}_{\partial _j\Omega _{{\hat{\jmath }}}})$ and every $K\in \mathcal {T}_{\Omega _{{\hat{\jmath }}}}$, the discrete lift satisfies

29 $$\begin{aligned} {\left( {\widetilde{\mathcal {R}}^*_j(w)\cdot {\boldsymbol{n}}_K},{1}\right) }_{\partial K} \;=\; {\left( {w},{1}\right) }_{\partial K\cap \partial _j\Omega _{{\hat{\jmath }}}}. \end{aligned}$$

Assumption 4 states that, on every bulk simplex *K*, the lifted function transports source-boundary data into the interior with no net flux contribution from any face of ∂K that does not lie on $\partial _j\Omega _{{\hat{\jmath }}}$. The discrete Raviart-Thomas-based lift constructed in [24] satisfies Assumption 4, since by construction it assigns zero face moments on every face of ∂K outside $\partial _j\Omega _{{\hat{\jmath }}}$, and in particular zero face mean. Other H(div)-conforming discrete lifts that share this structural property also fall within the scope of the framework.

#### Cellwise mass-defect property

The proof of Theorem 1 relies on a cellwise vanishing property of the grid-mismatch residual that follows from the mean-preserving construction of the discrete projections in Sect. 4.2.2 and from Assumption 4 on the discrete lift. We isolate this property in the following lemma, which we will invoke in Appendix A.

##### Lemma 3

(Cellwise mass-defect property of the grid-mismatch residual) Let ${\boldsymbol{v}} = [{\boldsymbol{v}}_{0, i}, \nu _j] \in \textbf{X}_h$ (a suitable finite-dimensional subspace of $\textbf{X}$) and define the grid-mismatch residual on each subdomain $\Omega _i$, i∈I, by

30 $$\begin{aligned} \rho _i \;:=\; \nabla _i\cdot \textstyle {\sum }_{j\in \check{S}_i}\widetilde{\mathcal {R}}^*_j\circ \delta \mathcal {D}_j^{{\hat{\jmath }}}\nu _j \;+\; \textstyle {\sum }_{j\in \hat{S}_i}\delta \mathcal {D}_j^{{\check{\jmath }}}\nu _j, \end{aligned}$$

where $\widetilde{\mathcal {R}}^*_j$ is interpreted on transfer-grid data via the convention (27). Then

31 $$\begin{aligned} {\left( {\rho _i},{1}\right) }_K \;=\; 0 \qquad \forall \, K\in \mathcal {T}_{\Omega _i},\ i\in I. \end{aligned}$$

##### Proof

The two contributions to $\rho _i$ are treated separately.

For the lower-dimensional term, we fix $j\in \hat{S}_i$, so that $i = \check{\jmath }$, and let $K\in \mathcal {T}_{\Omega _{{\check{\jmath }}}}$. By the mean-preserving property of the constrained $L^2$ projection $\widetilde{\mathcal {D}}_j^{{\check{\jmath }}}$ established in Lemma 2,

$$\begin{aligned} {\left( {\widetilde{\mathcal {D}}_j^{{\check{\jmath }}}\nu _j},{1}\right) }_K \;=\; \sum _{K_{\Gamma _j,\Omega _{{\check{\jmath }}}}\subset K}{\left( {\nu _j},{1}\right) }_{K_{\Gamma _j,\Omega _{{\check{\jmath }}}}} \;=\; {\left( {\mathcal {D}_j^{{\check{\jmath }}}\nu _j},{1}\right) }_K, \end{aligned}$$

where the second equality uses that the canonical $L^2$ identification $\mathcal {D}_j^{{\check{\jmath }}}\nu _j$ on $K\in \mathcal {T}_{\Omega _{{\check{\jmath }}}}$ is given by the integral of $\nu _j$ over the union of transfer cells $K_{\Gamma _j,\Omega _{{\check{\jmath }}}}\subset K$. Subtracting yields ${\left( {\delta \mathcal {D}_j^{{\check{\jmath }}}\nu _j},{1}\right) }_K = 0$.

For the bulk term, we fix $j\in \check{S}_i$, so that $i = \hat{\jmath }$, and let $K\in \mathcal {T}_{\Omega _{{\hat{\jmath }}}}$. By the convention (27), the lift acts on transfer-grid data through the constrained projection $\widetilde{\mathcal {D}}_{j,{\hat{\jmath }}}^{{\hat{\jmath }}}$, and Assumption 4 applied to the discrete data $w:= \widetilde{\mathcal {D}}_{j,{\hat{\jmath }}}^{{\hat{\jmath }}}\bigl (\delta \mathcal {D}_j^{{\hat{\jmath }}}\nu _j\bigr )\in \mathbb {P}_k(\mathcal {T}_{\partial _j\Omega _{{\hat{\jmath }}}})$ gives

$$\begin{aligned} {\left( {\nabla _i\cdot \widetilde{\mathcal {R}}^*_j\circ \delta \mathcal {D}_j^{{\hat{\jmath }}}\nu _j},{1}\right) }_K \;=\; \sum _{e\subset \partial K\cap \partial _j\Omega _{{\hat{\jmath }}}} {\left( {\widetilde{\mathcal {D}}_{j,{\hat{\jmath }}}^{{\hat{\jmath }}}\,\delta \mathcal {D}_j^{{\hat{\jmath }}}\nu _j},{1}\right) }_{e}. \end{aligned}$$

Each face $e\subset \partial K\cap \partial _j\Omega _{{\hat{\jmath }}}$ is an element of the internal boundary grid $\mathcal {T}_{\partial _j\Omega _{{\hat{\jmath }}}}$ (since the latter is the trace of $\mathcal {T}_{\Omega _{{\hat{\jmath }}}}$ on $\partial _j\Omega _{{\hat{\jmath }}}$), so the constrained $L^2$ projection $\widetilde{\mathcal {D}}_{j,{\hat{\jmath }}}^{{\hat{\jmath }}}$ preserves the face mean of its argument:

$$\begin{aligned} {\left( {\widetilde{\mathcal {D}}_{j,{\hat{\jmath }}}^{{\hat{\jmath }}}\,\delta \mathcal {D}_j^{{\hat{\jmath }}}\nu _j},{1}\right) }_e \;=\; {\left( {\delta \mathcal {D}_j^{{\hat{\jmath }}}\nu _j},{1}\right) }_e \;=\; {\left( {\mathcal {D}_j^{{\hat{\jmath }}}\nu _j},{1}\right) }_e - {\left( {\widetilde{\mathcal {D}}_j^{{\hat{\jmath }}}\nu _j},{1}\right) }_e \;=\; 0, \end{aligned}$$

where the last equality uses Lemma 2 (mean-preserving property of $\widetilde{\mathcal {D}}_j^{{\hat{\jmath }}}$ on $\mathcal {T}_{\partial _j\Omega _{{\hat{\jmath }}}}$). Summing over all faces $e\subset \partial K\cap \partial _j\Omega _{{\hat{\jmath }}}$ gives ${\left( {\nabla _i\cdot \widetilde{\mathcal {R}}^*_j\circ \delta \mathcal {D}_j^{{\hat{\jmath }}}\nu _j},{1}\right) }_K = 0$. Adding the two cellwise vanishing identities yields (31). □

## A posteriori error estimation

In this section, we focus on the a posteriori analysis on non-matching grids. We start by introducing the admissible mD functions that can be used to estimate the errors, then we present our main result: the *a posteriori* error bounds on non-matching grids. Finally, we introduce error indicators that will be employed in the numerical examples.

### Admissible mixed-dimensional pairs on non-matching grids

The functional estimates below are stated for comparison pairs that are conforming in the energy spaces, locally conservative on the non-matching grid, and compatible with the computable transfer operators entering the estimator. The construction of such pairs from particular numerical methods is discussed in the next section.

Let $Q_h^g\subset H^1_0(\Omega )+g$ be a finite-dimensional affine space of conforming mD potentials, and let $\textbf{X}_h\subset \textbf{X}$ be a finite-dimensional space of conforming mD fluxes, both associated with the non-matching mD partition of $Y=\Omega \sqcup \Gamma$. We assume that the restrictions and traces of functions in $Q_h^g$, and the interface components of functions in $\textbf{X}_h$, are compatible with the discrete projection and grid mismatch operators introduced in Sect. 4.

#### Definition 7

*(Locally conservative flux on a non-matching grid)* Let ${\boldsymbol{v}}=[{\boldsymbol{v}}_{0,i},\nu _j]\in \textbf{X}_h$. For each i∈I, define the non-matching residual

$$ \widetilde{r}_i({\boldsymbol{v}};f):= f_i - \nabla _i\cdot \left( {\boldsymbol{v}}_{0,i} + \textstyle {\sum }_{j\in \check{S}_i} {\widetilde{\mathcal {R}}}_j^* \circ {\widetilde{\mathcal {D}}}_j^{{\hat{\jmath }}}\nu _j \right) + \textstyle {\sum }_{j\in \hat{S}_i} {\widetilde{\mathcal {D}}}_j^{{\check{\jmath }}}\nu _j. $$

We say that ${\boldsymbol{v}}$ is locally conservative on the non-matching grid if

$$ \bigl (\widetilde{r}_i({\boldsymbol{v}};f),1\bigr )_K=0 \qquad \forall K\in \mathcal T_{\Omega _i},\quad \forall i\in I. $$

The corresponding admissible class is

$$ \textbf{X}_{h, \textrm{LC}}(f):= \left\{ {\boldsymbol{v}}\in \textbf{X}_h: \bigl (\widetilde{r}_i({\boldsymbol{v}};f),1\bigr )_K=0 \quad \forall K\in \mathcal T_{\Omega _i},\quad \forall i\in I \right\} . $$

Note that to satisfy Definition 7 on non-matching grids, the discrete projection operators $\widetilde{\mathcal {D}}_{j}^{{\hat{\jmath }}}$ and $\widetilde{\mathcal {D}}_{j}^{{\check{\jmath }}}$ (cf. Section 4.2.2) play a key role, as their construction guarantees inter-dimensional mass conservation.

#### Definition 8

*(Admissible mD pair on non-matching grids)* A pair $(q,{\boldsymbol{v}})\in Q_h^g\times \textbf{X}_h$ is called admissible on the non-matching grid if

$$ {{\boldsymbol{v}}}\in \textbf{X}_{h,\textrm{LC}}(f), $$

and all transferred, lifted, and defect quantities entering the majorant are well defined and finite. In particular, the quantities

$$\begin{aligned}{\widetilde{\mathcal {P}}}_{{\hat{\jmath }}}^j q_{{\hat{\jmath }}},\quad {\widetilde{\mathcal {P}}}_{{\check{\jmath }}}^j q_{{\check{\jmath }}},\quad \delta \mathcal {P}_{{\hat{\jmath }}}^j q_{{\hat{\jmath }}},\quad \delta \mathcal {P}_{{\check{\jmath }}}^j q_{{\check{\jmath }}}, \end{aligned}$$

and

$$\begin{aligned}{\widetilde{\mathcal {D}}}_{j}^{{\hat{\jmath }}}\nu _j,\quad {\widetilde{\mathcal {D}}}_{j}^{{\check{\jmath }}}\nu _j,\quad \delta \mathcal {D}_j^{{\hat{\jmath }}}\nu _j,\quad \delta \mathcal {D}_j^{{\check{\jmath }}}\nu _j \end{aligned}$$

are well defined for every coupling triplet, and the lifted defect fields and their elementwise divergences belong to the required $L^2$ spaces.

### Main results

The following theorem presents the main result of this work.

#### Theorem 1

(Guaranteed upper bounds on non-matching grids) For every $q \in Q_h^g$ and every ${\boldsymbol{v}}\in \textbf{X}_{h, \textrm{LC}}(f)$ in the sense of Definition 8, define the majorant as

32 $$\begin{aligned} \mathcal {M}(q, {\boldsymbol{v}}, f) := \eta _{\textrm{NM, R}}({\boldsymbol{v}}, f) + \eta _{\textrm{GM,R}}({\boldsymbol{v}}) + \eta _{\textrm{NM, DF}}(q, {\boldsymbol{v}}) + \eta _{\textrm{GM,DF}}(q, {\boldsymbol{v}}), \end{aligned}$$

with the global indicators

33a $$\begin{aligned} \eta _{\textrm{NM, R}}^2({\boldsymbol{v}}, f)&= \sum _{i\in I} \sum _{K\in \mathcal {T}_{\Omega _i}} \eta ^2_{\textrm{NM, R}, K}, \end{aligned}$$

33b $$\begin{aligned} \eta _{\textrm{GM,R}}^2({\boldsymbol{v}})&= \sum _{i\in I} \sum _{K\in \mathcal {T}_{\Omega _i}} \eta ^2_{\textrm{GM, R}, K}, \end{aligned}$$

33c $$\begin{aligned} \eta _{\textrm{NM, DF}}^2(q, {\boldsymbol{v}})&= \sum _{i\in I} \sum _{K\in \mathcal {T}_{\Omega _i}} \eta ^2_{\textrm{NM, DF}_\parallel , K} + \sum _{j\in J} \sum _{K\in \mathcal {T}_{\Gamma _j}} \eta ^2_{\textrm{NM, DF}_\perp , K}, \end{aligned}$$

33d $$\begin{aligned} \eta _{\textrm{GM,DF}}^2(q, {\boldsymbol{v}})&= \sum _{i\in I} \sum _{K\in \mathcal {T}_{\Omega _i}} \eta ^2_{\textrm{GM}, \textrm{DF}_\parallel , K} + \sum _{j\in J} \sum _{K\in \mathcal {T}_{\Gamma _j}} \eta ^2_{\textrm{GM}, \textrm{DF}_\perp , K}, \end{aligned}$$

and the local error estimators given by

34a $$\begin{aligned} \eta _{\textrm{NM, DF}_\parallel , K}&= \left\Vert \mathcal {K}_i^{-1/2}\left( {\boldsymbol{v}}_{0, i} + \textstyle {\sum }_{j\in \check{S}_i} \widetilde{\mathcal {R}}^*_j\circ \widetilde{\mathcal {D}}_j^{\hat{\jmath }}\nu _j\right) + \mathcal {K}_i^{1/2} \nabla _i q_i \right\Vert _K, \end{aligned}$$

34b $$\begin{aligned} \eta _{\textrm{NM, DF}_\perp , K}&= \left\Vert \kappa _j^{-1/2}\nu _j + \kappa _j^{1/2} \left( \widetilde{\mathcal {P}}_{\check{\jmath }}^j q_{\check{\jmath }}- \widetilde{\mathcal {P}}_{\hat{\jmath }}^j q_{\hat{\jmath }}\right) \right\Vert _K, \end{aligned}$$

34c $$\begin{aligned} \eta _{\textrm{NM, R}, K}&= \frac{h_K}{\pi c_{\mathcal {K}_i, K}}\left\Vert f_i- \nabla _i \cdot \left( {\boldsymbol{v}}_{0, i} + \textstyle {\sum }_{j\in \check{S}_i} \widetilde{\mathcal {R}}^*_j \circ \widetilde{\mathcal {D}}_j^{\hat{\jmath }}\nu _j \right) + \textstyle {\sum }_{j\in \hat{S}_i} \widetilde{\mathcal {D}}_j^{{\check{\jmath }}} \nu _j \right\Vert _K, \end{aligned}$$

34d $$\begin{aligned} \eta _{\textrm{GM}, \textrm{DF}_\parallel , K}&= \left\Vert \mathcal {K}_i^{-1/2}\textstyle {\sum }_{j\in \check{S}_i} \widetilde{\mathcal {R}}_j^* \circ \delta \mathcal {D}_j^{\hat{\jmath }}\nu _j \right\Vert _K, \end{aligned}$$

34e $$\begin{aligned} \eta _{\textrm{GM}, \textrm{DF}_\perp , K}&= \left\Vert \kappa _j^{1/2} \left( \delta \mathcal {P}^j_{\check{\jmath }}q_{\check{\jmath }}- \delta \mathcal {P}_{\hat{\jmath }}^j q_{{\hat{\jmath }}}\right) \right\Vert _K, \end{aligned}$$

34f $$\begin{aligned} \eta _{\textrm{GM}, \textrm{R}, K}&= \frac{h_K}{\pi c_{\mathcal {K}_i, K}} \left\Vert \nabla _i \cdot \textstyle {\sum }_{j\in \check{S}_i} \widetilde{\mathcal {R}}_j^* \circ \delta \mathcal {D}_j^{\hat{\jmath }}\nu _j + \textstyle {\sum }_{j\in \hat{S}_i} \delta \mathcal {D}_j^{\check{\jmath }}\nu _j \right\Vert _K. \end{aligned}$$

Then, the following estimates hold:

(i) Let $p=[p_i]\in H^1_0(\Omega )+g$ be the solution of Problem 3. Then
  35 $$\begin{aligned} {\left| \hspace{-1.0625pt}\left| \hspace{-1.0625pt}\left| p-q \right| \hspace{-1.0625pt}\right| \hspace{-1.0625pt}\right| } \le \mathcal {M}(q, {\boldsymbol{v}}, f) =: \mathcal {M}^{\oplus }_p \qquad \forall {\boldsymbol{v}}\in \textbf{X}_{h, \textrm{LC}}(f). \end{aligned}$$
(ii) Let ${\boldsymbol{u}}=[{\boldsymbol{u}}_{0,i},\lambda _j]\in \textbf{X}$ be the solution of Problem 4. Then
  36 $$\begin{aligned} {\left| \hspace{-1.0625pt}\left| \hspace{-1.0625pt}\left| {\boldsymbol{u}}-{\boldsymbol{v}} \right| \hspace{-1.0625pt}\right| \hspace{-1.0625pt}\right| }_* \le \mathcal {M}(q, {\boldsymbol{v}}, f) =: \mathcal {M}^{\oplus }_{{\boldsymbol{u}}} \qquad \forall q\in Q_h^g. \end{aligned}$$

The proof follows the abstract functional framework of [20, 34, 35] adapted to the non-matching mD setting: we expand the energy norm of the error using the weak primal form, apply the mD integration-by-parts formula of [1] on the admissible pair $(p-q,{\boldsymbol{v}})\in H_0^1(\Omega )\times \textbf{X}_{h, \textrm{LC}}(f)$, and split each continuous transfer operator into its discrete realization plus a grid-mismatch defect. Cellwise Cauchy–Schwarz and the Poincaré–Wirtinger inequality (the latter applicable thanks to the local mass-conservation property of ${\boldsymbol{v}}\in \textbf{X}_{h, \textrm{LC}}(f)$ together with the cellwise mass-defect property of the discrete grid-mismatch operators) then yield the four global indicators in (32). For completeness, the full proof is given in Appendix A.

#### Remark 9

*(Nature of the estimates)* The majorant of Theorem 1 consists of two qualitatively different parts. The terms $\eta _{\textrm{NM, DF}_\parallel ,K}$, $\eta _{\textrm{NM, DF}_\perp ,K}$, and $\eta _{\textrm{NM, R},K}$ are the diffusive and residual contributions obtained from the admissible pair after non-matching transfer and mimic the error indicators from [20] in structure. The additional terms $\eta _{\textrm{GM}, \textrm{DF}_\parallel ,K}$, $\eta _{\textrm{GM}, \textrm{DF}_\perp ,K}$, and $\eta _{\textrm{GM}, \textrm{R},K}$ quantify the discrepancy introduced by evaluating the estimator on geometrically incompatible neighboring grids.

#### Remark 10

*(Behavior of grid mismatch indicators at lowest order)* For dual approximations with k=0, the bulk grid-mismatch indicator $\eta _{\textrm{GM}, \textrm{DF}_\parallel ,K}$ and the higher-dimensional contribution to $\eta _{\textrm{GM},\textrm{R},K}$ vanish identically, since at k=0 the constrained $L^2$ projection $\widetilde{\mathcal {D}}_{j,{\hat{\jmath }}}^{{\hat{\jmath }}}$ is the cell average and $\delta \mathcal {D}_j^{{\hat{\jmath }}}\nu _j$ has zero face mean on every face of $\mathcal {T}_{\partial _j\Omega _{{\hat{\jmath }}}}$. The interface mismatch $\eta _{\textrm{GM}, \textrm{DF}_\perp ,K}$ and the lower-dimensional contribution to $\eta _{\textrm{GM}, \textrm{R},K}$ remain nontrivial, since they capture the loss of higher moments by the Scott–Zhang projection on $\Gamma _j$ and by the constrained $L^2$ projection on $\mathcal {T}_{\Omega _{{\check{\jmath }}}}$, respectively.

#### Corollary 1

*(Sharpness under grid compatibility)* In the case when the mixed-dimensional grid is matching, $\mathcal {P}_\bullet ^j = \widetilde{\mathcal {P}}_\bullet ^j$ and $\mathcal {D}_j^\bullet =\widetilde{\mathcal {D}}_j^\bullet$, which implies zero defect and consequently $\eta _{\textrm{GM}, \textrm{R}, K} = \eta _{\textrm{GM}, \textrm{DF}_\parallel , K} = \eta _{\textrm{GM}, \textrm{DF}_\perp , K} = 0$. In this case, the majorant reduces to its standard form $\mathcal {M}(q, {\boldsymbol{v}}, f) = \eta _{\textrm{DF}}(q, {\boldsymbol{v}}) + \eta _{\textrm{R}}({\boldsymbol{v}}, f)$ which matches with the error bounds for locally-mass conservative approximations on matching grids from [20, Sec. 5.2.3].

Corollary 1 justifies the claim that the present work is an extension of our previous results [20] on matching grids.

### Error indicators

It is often desirable to associate error estimates with an entire subdomain or interface grid. To this aim, we introduce the subdomain- and interface-level non-matching and grid-mismatch error indicators

37 $$\begin{aligned} \eta _{\textrm{NM},\Omega _i}^2&:= \sum _{K\in \mathcal {T}_{\Omega _i}} \left( \eta _{\textrm{NM},\textrm{DF}_{\parallel },K}^2 + \eta _{\textrm{NM},\textrm{R},K}^2 \right) ,&\eta _{\textrm{NM},\Gamma _j}^2&:= \sum _{K\in \mathcal {T}_{\Gamma _j}} \eta _{\textrm{NM},\textrm{DF}_{\perp },K}^2, \end{aligned}$$

38 $$\begin{aligned} \eta _{\textrm{GM},\Omega _i}^2&:= \sum _{K\in \mathcal {T}_{\Omega _i}} \left( \eta _{\textrm{GM},\textrm{DF}_{\parallel },K}^2 + \eta _{\textrm{GM},\textrm{R},K}^2 \right) ,&\eta _{\textrm{GM},\Gamma _j}^2&:= \sum _{K\in \mathcal {T}_{\Gamma _j}} \eta _{\textrm{GM},\textrm{DF}_{\perp },K}^2. \end{aligned}$$

The total indicators associated with each subdomain and interface are

39 $$\begin{aligned} \eta _{\Omega _i}^2 := \eta _{\textrm{NM},\Omega _i}^2 + \eta _{\textrm{GM},\Omega _i}^2, \qquad \eta _{\Gamma _j}^2 := \eta _{\textrm{NM},\Gamma _j}^2 + \eta _{\textrm{GM},\Gamma _j}^2. \end{aligned}$$

To report results for complex fracture networks, it is beneficial to aggregate subdomain and interface errors of the same dimensionality. Let $I_{d_i}\subseteq I$ and $J_{d_j}\subseteq J$ be the index sets of subdomains and interfaces of dimensions $d_i$ and $d_j$, respectively. We then use

40 $$\begin{aligned} \eta _{\Omega ^{d_i}}^2 := \sum _{i\in I_{d_i}} \eta _{\Omega _i}^2, \qquad \eta _{\Gamma ^{d_j}}^2 := \sum _{j\in J_{d_j}} \eta _{\Gamma _j}^2, \end{aligned}$$

to denote the indicators associated with all $d_i$-dimensional subdomains and all $d_j$-dimensional interfaces, respectively.

## Computable estimates

Theorem 1 is independent of a particular numerical method, provided that the admissible pair belongs to $Q_h^g\times \textbf{X}_{h,\textrm{LC}}(f)$. We now describe two concrete realizations, based on the primal and dual weak forms from Sect. 3.3.

### Primal and dual approximations

With the discrete projection operators formally defined, we can now write the approximated primal weak and dual problems. To this aim, consider first the mD broken space of polynomials on subdomains of order *k* or less:

41 $$\begin{aligned} \mathbb {P}_k(\mathcal {T}_{\Omega }) := \prod _{i\in I} \mathbb {P}_k(\mathcal {T}_{\Omega _{i}}). \end{aligned}$$

#### Problem 5

(Approximated weak primal form on non-matching grids) Let k≥1 be fixed and $p_h=[p_{h, i}]$, $g = [g_i] \in H^1(\Omega )$. Then, given $f = [f_i] \in L^2(\Omega )$, find $p_h \in \mathbb {P}_k(\mathcal {T}_{\Omega }) \cap ( H^1_0(\Omega ) + g)$ such that

42 $$\begin{aligned}&\sum _{i\in I} \sum _{K\in \mathcal {T}_{\Omega _{i}}}\left( \mathcal {K}_i\nabla _i{p_{h,i}}, \nabla _iq_{h,i}\right) _{K} + \sum _{j\in J}\sum _{K\in \mathcal {T}_{\Gamma _{j}}} \left( \kappa _j( \widetilde{\mathcal {P}}_{{\check{\jmath }}}^{j}p_{h,{\check{\jmath }}} - \widetilde{\mathcal {P}}_{{\hat{\jmath }}}^{j}p_{h, {\hat{\jmath }}}), \widetilde{\mathcal {P}}_{{\check{\jmath }}}^{j}q_{h, {\check{\jmath }}} - \widetilde{\mathcal {P}}_{{\hat{\jmath }}}^{j}q_{h, {\hat{\jmath }}}\right) _{K} \nonumber \\&~~~~=\sum _{i\in I} \sum _{K\in \mathcal {T}_{\Omega _{i}}}\left( f_i, q_{h,i}\right) _{K} \qquad \forall q_h=[q_{h, i}]\in \mathbb {P}_k(\mathcal {T}_\Omega )\cap H^1_0(\Omega ). \end{aligned}$$

Consider now the dual approximated problem. We wish to construct the discrete space for the mD fluxes, i.e., the discrete counterpart of $\textbf{X}$. To this aim, we first consider the local Raviart-Thomas space given by

43 $$\begin{aligned} \mathbb{R}\mathbb{T}_k(\mathcal {T}_{\Omega _{i}}) = \{{\boldsymbol{v}}_{h, i}\in \textbf{H}(\textrm{div},\Omega _i): {\boldsymbol{v}}_{h, i}|_{K}\in \mathbb{R}\mathbb{T}_k(K) ~\forall ~K\in \mathcal {T}_{\Omega _{i}}\}. \end{aligned}$$

Then, the space $\textbf{X}_k$ is given by

44 $$\begin{aligned} \textbf{X}_k = \prod _{i\in I} \left[ \left( \mathbb{R}\mathbb{T}_k(\mathcal {T}_{\Omega _{i}}) \cap \textbf{V}_{0,i}\right) \times \prod _{j\in \check{S}_i}\widetilde{\mathcal {R}}^*_j\circ \widetilde{\mathcal {D}}_{j}^{{\hat{\jmath }}}\,\mathbb {P}_k(\mathcal {T}_{\Gamma _{j}})\right] , \end{aligned}$$

where $\widetilde{\mathcal {R}}^*_j: \mathbb {P}_k(\mathcal {T}_{\partial _{j}\Omega _{{\hat{\jmath }}}})\rightarrow \mathbb{R}\mathbb{T}_k(\mathcal {T}_{\Omega _{{\hat{\jmath }}}})$ is the discrete extension operator from internal boundary grids onto the interior of higher-dimensional subdomain grids, cf. [13, 24] for a detailed discussion of the construction of this operator. We can now write the approximated dual weak problem as follows.

#### Problem 6

(Approximated weak dual form on non-matching grids) Let k≥0 and ${\boldsymbol{u}}_h = [{\boldsymbol{u}}_{h, 0, i}, \lambda _{h, j}]$, $p_h = [p_{h, i}]$. Then, given $f=[f_i]\in L^2(\Omega )$ and $g_D = [g_{D,i}] \in H^{1/2}(\partial _D\Omega )$, find ${\boldsymbol{u}}_h \in \textbf{X}_{k}$ and $p_h\in \mathbb {P}_k(\mathcal {T}_\Omega )$ such that

45a $$\begin{aligned}&\sum _{i\in I} \sum _{K\in \mathcal {T}_{\Omega _{i}}}{\left( {\mathcal {K}^{-1}_i\left( {\boldsymbol{u}}_{h,0,i} + \textstyle {\sum }_{j\in \check{S}_i}\widetilde{\mathcal {R}}^*_j\circ \widetilde{\mathcal {D}}_{j}^{{\hat{\jmath }}}\lambda _{h,j}\right) },{{\boldsymbol{v}}_{h, 0,i} + \textstyle {\sum }_{j\in \check{S}_i}\widetilde{\mathcal {R}}^*_j\circ \widetilde{\mathcal {D}}_{j}^{{\hat{\jmath }}}\nu _{h,j}}\right) }_{K} \nonumber \\&~~\quad - \sum _{i\in I} \sum _{K\in \mathcal {T}_{\Omega _{i}}}{\left( {p_{h, i}},{\nabla _i\cdot \left( {\boldsymbol{v}}_{h, 0,i}+\textstyle {\sum }_{j\in \check{S}_i}\widetilde{\mathcal {R}}^*_j\circ \widetilde{\mathcal {D}}_{j}^{{\hat{\jmath }}}\nu _{h, j}\right) + \textstyle {\sum }_{j\in \hat{S}_i}\widetilde{\mathcal {D}}_{j}^{{\check{\jmath }}}\nu _{h,j}}\right) }_{K} \nonumber \\&~~\quad +\sum _{j\in J} \sum _{K\in \mathcal {T}_{\Gamma _{j}}}{\left( {\kappa _j^{-1}\lambda _{h,j}},{\nu _{h,j}}\right) }_{K} \nonumber \\&~~= -\sum _{i\in I} \sum _{K\in \mathcal {T}_{\partial _D\Omega _i}} {\left( {g_{D,i}},{\left( {\boldsymbol{v}}_{h, 0,i} \cdot {\boldsymbol{n}}_i\right) |_K}\right) }_{K} \qquad \forall {\boldsymbol{v}}_h=[{\boldsymbol{v}}_{h, 0, i}, \nu _{h, j}] \in \textbf{X}_k. \end{aligned}$$

45b $$\begin{aligned}&\sum _{i\in I} \sum _{K\in \mathcal {T}_{\Omega _{i}}}{\left( {\nabla _i\cdot \left( {\boldsymbol{u}}_{h, 0,i} + \textstyle {\sum }_{j\in \check{S}_i}\widetilde{\mathcal {R}}^*_j\circ \widetilde{\mathcal {D}}_{j}^{{\hat{\jmath }}}\lambda _{h, j}\right) },{q_{h, i}}\right) }_{K} \nonumber \\&\quad ~~ - \sum _{i\in I} \sum _{K\in \mathcal {T}_{\Omega _{i}}} {\left( {\textstyle {\sum }_{j\in \hat{S}_i}\widetilde{\mathcal {D}}_{j}^{{\check{\jmath }}}\lambda _{h, j}},{q_{h, i}}\right) }_{K} \nonumber \\&~~=\sum _{i\in I} \sum _{K\in \mathcal {T}_{\Omega _{i}}} {\left( {f_i},{q_{h, i}}\right) }_{K}\qquad \forall q_h=[q_{h, i}]\in \mathbb {P}_k(\mathcal {T}_{\Omega }). \end{aligned}$$

In Problem 6, the Dirichlet boundary grid $\mathcal {T}_{\partial _D\Omega _i}$ is defined in analogy to (4.1).

#### Remark 11

*(Stability and relative refinement of interface grids)* It is well known that classical mortar methods require $\mathcal {T}_{\Gamma _j}$ to be coarser or equal than $\mathcal {T}_{\partial _j\Omega _{\hat{\jmath }}}$. As shown in [9], the introduction of a diffusive problem in the fractures guarantees uniqueness of the solution even when this is not the case. Nevertheless, as pointed out in [24], to avoid possible oscillations in the interface flux variable $\lambda _{h, j}$, it is desirable to adopt $\mathcal {T}_{\Gamma _j}$ coarser or equal than $\mathcal {T}_{\partial _j\Omega _{\hat{\jmath }}}$ in practice.

### Conforming fluxes and potentials

In order to apply the *a posteriori* bounds from Theorem 1, we need an mD potential in $Q_h^g$ and an mD flux in $\textbf{X}_{h, \textrm{LC}}(f)$. Problem 5 only provides the first ingredient, whereas Problem 6 only provides the second ingredient. Thus, to obtain computable bounds using the finite element method one has to reconstruct the associated fluxes, while the mixed-finite element requires the reconstruction of potentials. Deriving optimal reconstruction techniques, even in the lowest-order case, is non-trivial, as one has to balance accuracy with computational cost. We therefore maintain the definition of conforming fluxes and potentials as general as possible while providing references to guide the reader.

#### Definition 9

*(Conforming flux)* We define a conforming flux as any function ${\boldsymbol{\sigma }}_h$ that satisfies

46 $$\begin{aligned} {\boldsymbol{\sigma }}_h \in \textbf{X}_{h, \textrm{LC}}(f). \end{aligned}$$

#### Remark 12

*(On flux reconstruction)* Classical flux reconstruction techniques include (i) *superconvergent patch recovery* [36, 37] with $H(\text {div})$-projections, (ii) local Neumann-patch solves [38–41], and (iii) hybrid explicit-recovery approaches [42]. In the mD setting, flux equilibration has been limited to enforcing mass balance across 2D/1D matrix–fracture interfaces couplings [17, 18, 43]. To our knowledge, a general mD flux reconstruction framework has not yet been proposed.

#### Definition 10

*(Conforming potential)* We define a conforming potential as any function $s_h$ that satisfies

47 $$\begin{aligned} s_h \in Q_h^g. \end{aligned}$$

#### Remark 13

*(On potential reconstruction)* Several techniques exist to recover continuous potentials from a non-conforming discretization in individual subdomains. Common approaches include: (i) patch-wise weighted average of cell-centered potentials (e.g., Green-Gauss, distance-weighted averages and least squares fits) [44–48], (ii) element-wise quadratic reconstruction via local polynomial solves [49, 50], and (iii) element-wise linear postprocessing based on lowest-order Raviart-Thomas basis functions [51]. Since the mD formulation does not require inter-dimensional continuity of potentials, any of these methods can be applied independently to each subdomain grid.

### Fully computable a posteriori error estimates

Computable estimates are now readily available for primal and dual approximations.

#### Definition 11

*(Computable bounds for finite element approximations)* Let $p_h\in \mathbb {P}_k(\mathcal {T}_{\Omega })\cap (H^1_0(\Omega )+ g)$ with k≥1 be the solution to Problem 5, and ${\boldsymbol{\sigma }}_h\in \textbf{X}_{h, \textrm{LC}}(f)$ for k≥0 be a conforming flux in the sense of Definition 9. Then, set $q=p_h$ and ${\boldsymbol{v}}={\boldsymbol{\sigma }}_h$ in Theorem 1 to obtain fully computable bounds.

#### Definition 12

*(Computable bounds for mixed-finite element approximations)* Let $({\boldsymbol{u}}_h, p_h) \in \textbf{X}_k\times \mathbb {P}_k(\mathcal {T}_\Omega )$ with k≥0 be the solution to Problem 6, and let $s_h\in \mathbb {P}_k(\mathcal {T}_\Omega ) \cap (H^1_0(\Omega )+ g)$ for k≥1 be a conforming potential in the sense of Definition 10. Then, set $q=s_h$ and ${\boldsymbol{v}}={\boldsymbol{u}}_h$ in Theorem 1 to obtain fully computable bounds.

Thanks to the functional-based construction of the bounds in Theorem 1 and provided that approximate potentials and fluxes are properly reconstructed, other classical discretization techniques also fall within our framework. Important methods used in flow modeling in fractured porous media include the cell-centered finite volume method (CCFVM) [52, 53]—both the two-point flux approximation (TPFA) and the multi-point flux approximation (MPFA)—, the Vertex-Centered Finite Volume Method [54], the Mimetic Finite Difference Method [55, 56], the Discontinuous Galerkin Method [57, 58], and the Dual Mixed Virtual Element Method (MVEM) [12].

### Effectivity indices

To quantify the efficiency of the *a posteriori* error estimates, we shall employ the following effectivity indices

48 $$\begin{aligned}&I^{\text {eff}}_p = \frac{\mathcal {M}_p^{\oplus }(s_h, {\boldsymbol{\sigma }}_h,f)}{{\left| \hspace{-1.0625pt}\left| \hspace{-1.0625pt}\left| p-s_h \right| \hspace{-1.0625pt}\right| \hspace{-1.0625pt}\right| }}, \qquad \nonumber \\&I^{\text {eff}}_{{\boldsymbol{u}}} = \frac{\mathcal {M}_{{\boldsymbol{u}}}^\oplus (s_h, {\boldsymbol{\sigma }}_h, f)}{{\left| \hspace{-1.0625pt}\left| \hspace{-1.0625pt}\left| {\boldsymbol{u}}-{\boldsymbol{\sigma }}_h \right| \hspace{-1.0625pt}\right| \hspace{-1.0625pt}\right| }_*}. \end{aligned}$$

## Numerical examples

In this section, we present numerical verifications of the proposed non-matching error estimators against manufactured solutions and their application for test cases based on community benchmarks of flow in fractured porous media. For the sake of compactness, we only perform our analysis on mD domains with 3D hosts given that cases with 2D hosts are considerably less complex.

To obtain the numerical approximations, we employ the MPFA method [25, 52, 53], which is closely related to the lowest-order mixed-finite element approximation [59]. Fully computable estimates are obtained after two postprocessing steps:

(i) MPFA face fluxes are directly mapped to the coefficients of the local RT0 space (guaranteeing continuity of normal traces) [44]. Repeating this process in each subdomain grid yields a conforming mD flux ${\boldsymbol{\sigma }}_h \in \textbf{X}_0$ (cf. Definition 9).
(ii) MPFA potentials are reconstructed in each subdomain grid to obtain a conforming mD potential $s_h \in \mathbb {P}_1(\mathcal {T}_\Omega )\cap (H^1_0(\Omega )+g)$ using the potential reconstruction technique proposed in [51]. The technique employs the local MPFA face fluxes to first approximate a cell-centered potential gradient at the cell’s barycenter which is then mapped to the cell vertices and added to the cell-center potential. This process produces $\mathbb {P}_1$-nodal potentials which are generally non-conforming. Thus, the Oswald interpolator is applied as a final step to produce a conforming global linear potential $s_h \in H^1_0(\Omega ) + g$ (cf. Definition 10).

All numerical examples were implemented in PorePy [25] and are available for reproduction through the extension package mdnme [60].

**Fig. 5:**
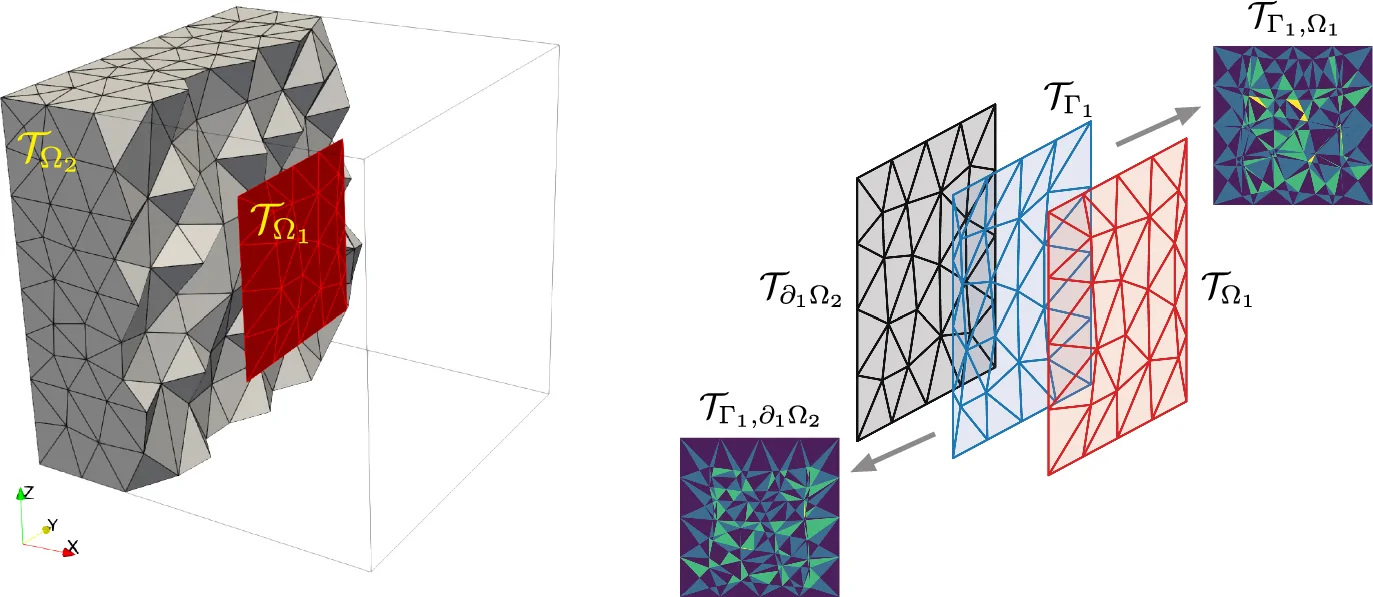
3D/2D numerical verification setup. Left: Mixed-dimensional grid showcasing the matrix grid $\mathcal {T}_{\Omega _2}$ and the fully embedded fracture grid $\mathcal {T}_{\Omega _1}$. Right: Exploded view of the non-matching coupling between $\mathcal {T}_{\partial _1\Omega _2}$ (black), $\mathcal {T}_{\Gamma _1}$ (blue), and $\mathcal {T}_{\Omega _1}$ (red) with the corresponding transfer grids $\mathcal {T}_{\Gamma _1, \partial _1\Omega _2}$ and $\mathcal {T}_{\Gamma _1, \Omega _1}$ (triangles are colored for visualization purposes only). Example shown for h=0.15 and translation $+ {\boldsymbol{e}}_y$

### 3D/2D verification with a manufactured solution

We employ the 3D/2D manufactured solution from Section 7 of [20] to verify the efficiency of the proposed error estimators. The mD domain consists of a unit cube $\Omega _2=(0,1)^3$ hosting a fully embedded vertical fracture

$$ \Omega _1=\{(x,y,z)\in \mathbb {R}^3:\ x=0.5,\ 0.25\le y\le 0.75,\ 0.25\le z\le 0.75\}. $$

Interfaces $\Gamma _1$ and $\Gamma _2$ couple $\Omega _2$ to the left and right sides of $\Omega _1$, respectively (see Fig. 5). With a smooth matrix pressure $p_2:\Omega _2\rightarrow \mathbb {R}$, unit tangential permeabilities $\mathcal {K}_2=\textbf{I}_{3\times 3}$ and $\mathcal {K}_1=\textbf{I}_{2\times 2}$, the exact Darcy fluxes ${\boldsymbol{u}}_2:\Omega _2\rightarrow \mathbb {R}^3$ and ${\boldsymbol{u}}_1:\Omega _1\rightarrow \mathbb {R}^2$ follow from (2b). With unit normal permeabilities $\kappa _1=\kappa _2=1$, the interface fluxes $\lambda _1:\Gamma _1\rightarrow \mathbb {R}$ and $\lambda _2:\Gamma _2\rightarrow \mathbb {R}$ follow from (2c). Finally, (2a) gives the exact sources $f_2:\Omega _2\rightarrow \mathbb {R}$ and $f_1:\Omega _1\rightarrow \mathbb {R}$. Exact expressions are listed in Appendix D.2 of [20].

We consider four refinement levels with target mesh sizes $h\in \{0.3,\,0.15,\,0.075, 0.0375\}$. For each *h*, we first build a fully matching tessellation of the mD grid (reference configuration). From this, we generate non-matching grids by systematically perturbing internal nodes of the fracture grid $\mathcal {T}_{\Omega _1}$ and the interface side grids $\mathcal {T}_{\Gamma _1}$ and $\mathcal {T}_{\Gamma _2}$. Concretely, each internal node of $\mathcal {T}_{\Omega _1}$ is translated by half of the grid’s mean cell diameter in the eight in-plane directions $\{\pm {\boldsymbol{e}}_y,\pm {\boldsymbol{e}}_z,\pm ({\boldsymbol{e}}_y+{\boldsymbol{e}}_z),\pm ({\boldsymbol{e}}_y-{\boldsymbol{e}}_z)\}$, while internal nodes of $\mathcal {T}_{\Gamma _1}$ and $\mathcal {T}_{\Gamma _2}$ are perturbed in the opposite directions (same magnitude). Nodes of the internal boundary grids $\mathcal {T}_{\partial _1\Omega _2}$ and $\mathcal {T}_{\partial _2\Omega _2}$ are left unperturbed. This procedure yields eight fully non-matching configurations per *h*; see the right panel of Fig. 5 for the left non-matching coupling corresponding to h=0.15 and $+{\boldsymbol{e}}_y$ perturbation direction.

Table 1 reports the subdomain and interface-wise non-matching and grid-mismatch error indicators together with the majorants, true errors, and effectivity indices for both matching (M) and non-matching (NM) realizations. For the non-matching results we report the mean over the eight perturbations together with the sample standard deviation. The results indicate that: (i) grid mismatch terms are about one order of magnitude smaller than their corresponding non-matching terms, (ii) majorants and effectivity indices for the primal and dual variables remain close to the matching baseline, and (iii) subdomain and interface contributions decrease monotonically with refinement. Moreover, as indicated in Remark 10, $\eta _{\textrm{GM}, \textrm{DF}_{\parallel }, K}$ is exactly zero (up to machine precision) and therefore not included in the table. It is worth emphasizing that the tested non-matching couplings used in this example are deliberately challenging (fully non-matching with finite perturbations) to stress-test the estimators; in applications, controlled refinement typically leads to even milder mismatches.

As can be verified in Table 1, the errors associated to the bulk subdomain $\Omega _2$ are dominant in all realizations. To show the potential of disjoint adaptivity guided by the error indicators, we complement the previous test with an additional experiment. In this experiment, we identify the subdomain with the highest error and uniformly refine it (via simplex bisection [61]) while keeping all the other grids fixed. During this process, non-matching grids are produced. The results are shown in Table 2 for four refinement steps. We can see that the error in the bulk monotonically decreases with refinement. However, due to the highly non-matching coupling, errors for the fracture and interfaces do not show a consistent decrease. Nevertheless, the estimators remain reliable for both pressure and flux variables as can be verified from the efficiency indices.

**Table 1:** Verification of the 3D/2D manufactured solution

	h=0.3		h=0.15		h=0.075		h=0.0375	
	M	NM	M	NM	M	NM	M	NM
$\eta _{\textrm{NM},\Omega _2}$	2.49e−01	2.49e−01 ± 1.61e−04	1.77e−01	1.77e−01 ± 1.39e−04	9.21e−02	9.20e−02 ± 7.44e−05	4.79e−02	4.80e−02 ± 4.09e−05
$\eta _{\textrm{NM},\Omega _1}$	8.56e−03	5.71e−03 ± 6.09e−03	1.63e−05	3.28e−03 ± 3.49e−03	2.92e−03	1.81e−03 ± 1.92e−03	5.31e−06	8.77e−04 ± 9.34e−04
$\eta _{\textrm{NM},\Gamma _1}$	7.04e−03	2.74e−02 ± 8.05e−03	2.29e−03	2.41e−02 ± 3.31e−03	4.69e−04	4.25e−03 ± 1.06e−03	7.94e−05	2.63e−03 ± 1.25e−03
$\eta _{\textrm{NM},\Gamma _2}$	5.56e−03	2.23e−02 ± 2.38e−03	2.46e−03	2.46e−02 ± 3.37e−03	4.61e−04	4.20e−03 ± 1.08e−03	8.08e−05	2.61e−03 ± 1.29e−03
$\eta _{\textrm{GM},\Gamma _1}$	–	2.59e−03 ± 4.35e−04	–	8.13e−04 ± 1.26e−04	–	7.86e−05 ± 7.14e−06	–	1.81e−05 ± 3.27e−06
$\eta _{\textrm{GM},\Gamma _2}$	–	1.21e−03 ± 2.51e−04	–	8.54e−04 ± 1.21e−04	–	8.21e−05 ± 9.12e−06	–	1.77e−05 ± 3.05e−06
$\eta _{\textrm{GM},\Omega _1}$	–	4.37e−05 ± 1.66e−05	–	1.43e−05 ± 1.14e−05	–	4.03e−06 ± 3.94e−06	–	9.60e−07 ± 1.02e−06
$\mathcal {M}^{\oplus }_p=\mathcal {M}^{\oplus }_{{\boldsymbol{u}}}$	2.75e−01	2.77e−01 ± 1.56e−03	1.93e−01	1.97e−01 ± 1.18e−03	9.66e−02	9.68e−02 ± 2.84e−04	4.92e−02	4.94e−02 ± 2.10e−04
${\left| \hspace{-1.0625pt}\left| \hspace{-1.0625pt}\left| p-p_h \right| \hspace{-1.0625pt}\right| \hspace{-1.0625pt}\right| }$	2.40e−01	2.43e−01 ± 7.76e−04	1.71e−01	1.75e−01 ± 4.50e−04	8.85e−02	8.82e−02 ± 4.86e−04	4.55e−02	4.64e−02 ± 8.82e−04
${\left| \hspace{-1.0625pt}\left| \hspace{-1.0625pt}\left| {\boldsymbol{u}}-{\boldsymbol{\sigma }}_h \right| \hspace{-1.0625pt}\right| \hspace{-1.0625pt}\right| }_*$	7.20e−02	7.16e−02 ± 4.17e−04	4.94e−02	5.01e−02 ± 7.14e−04	3.16e−02	3.05e−02 ± 1.26e−03	1.54e−02	1.75e−02 ± 2.23e−03
$I^{\textrm{eff}}_p$	1.15e+00	1.14e+00 ± 6.94e−03	1.13e+00	1.13e+00 ± 5.16e−03	1.09e+00	1.10e+00 ± 3.01e−03	1.08e+00	1.07e+00 ± 1.58e−02
$I^{\textrm{eff}}_{{\boldsymbol{u}}}$	3.82e+00	3.88e+00 ± 1.75e−02	3.91e+00	3.93e+00 ± 4.95e−02	3.05e+00	3.18e+00 ± 1.23e−01	3.19e+00	2.87e+00 ± 3.53e−01

Subdomain and interface error indicators, global majorants $\mathcal {M}^\oplus _p = \mathcal {M}^\oplus _{{\boldsymbol{u}}}$, true errors in pressure and flux, and effectivity indices. Column M: single matching-grid run; column NM: mean ± std over 8 mortar-translation directions

**Table 2:** Guided subdomain halving for the 3D/2D benchmark

$\text {Step}$	$|\mathcal {T}_{\Omega _2}|$	$|\mathcal {T}_{\Omega _1}|$	$\eta _{\Omega ^3}$	$\eta _{\Omega ^2}$	$\eta _{\Gamma ^2}$	$\mathcal {M}^{\oplus }_p=\mathcal {M}^{\oplus }_{{\boldsymbol{u}}}$	$I_{\textrm{eff},p}$	$I_{\textrm{eff},{{\boldsymbol{u}}}}$
0	941	14	2.56e−01	5.23e−05	1.10e−02	2.83e−01	1.145	3.826
1	7,528	14	1.43e−01	2.42e−05	1.54e−02	1.52e−01	1.098	3.723
2	60,224	14	7.25e−02	2.97e−05	2.22e−04	7.46e−02	1.072	3.576
3	481,792	14	3.64e−02	3.11e−05	8.77e−04	3.70e−02	1.058	3.539

At each step the subdomain with the larger indicator ($\eta _{\Omega ^3}$ or $\eta _{\Omega ^2}$) is bisected independently while the other remains fixed; the configuration is non-matching from step 1 onward. $|\mathcal {T}_{\Omega _2}|$ and $|\mathcal {T}_{\Omega _1}|$ denote the cell counts in the bulk and fracture subdomains, respectively

### Regular fracture network

**Fig. 6:**
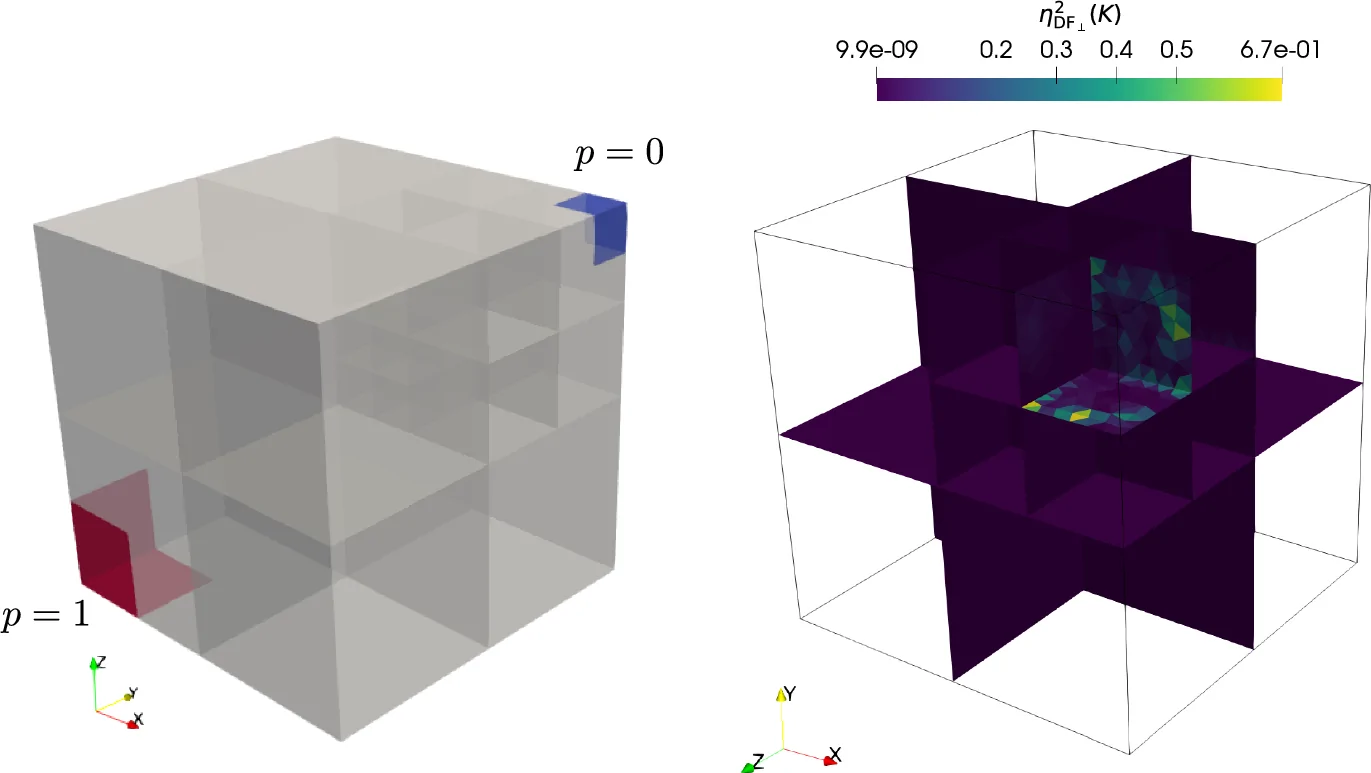
Regular network. Left: Geometry of the fracture network and boundary conditions. Right: Local diffusive error estimators of 2D interface grids for the finest mesh resolution

This numerical example employs the setup of Case 2: Regular network, from the 3D flow benchmark [7]. The mD domain consists of a unit cube matrix hosting nine regularly oriented fractures (see the left panel of Fig. 6), resulting in an mD domain with one 3D subdomain, nine 2D subdomains, sixty-nine 1D subdomains, and twenty-seven 0D intersection points.

The original benchmark prescribes no-flux boundary conditions in all external faces except on the red and blue regions, where a constant Darcy flux (${\boldsymbol{u}}=-1$) and a constant pressure (p=1) are imposed, respectively. Since our theory does not cover non-zero external boundary fluxes, and following [20], we replace the inflow Neumann condition by a Dirichlet condition with p=1 and set the outlet pressure to p=0. The matrix subdomain is assigned permeability values $\mathcal {K}\in \{1, 0.1\}$, while the fractures are considered conductive ($\mathcal {K} = 1\times 10^{4}$). The complete set of parameters can be found in Table 4 from [7].

The benchmark employs three levels of refinement, with approximately 500, 1400 and 32000 three-dimensional cells. To generate the non-matching grids, we begin from a fully matching tessellation of the mD domain and then globally refine all fracture grids using nested bisection [61]. Importantly, we do not refine the 1D subdomains grids. Refining 1D subdomain grids increases the resolution around intersection points—well-known points of singularity—without improving the accuracy of interface fluxes, resulting in a larger mismatch between the interface fluxes and the (permeability-scaled) pressure jumps (2c). We emphasize that this phenomenon is related to the limited regularity of the model itself [3], rather than to any deficiency of the error estimators.

Table 3 reports the aggregated local errors grouped by dimensionality, together with the majorant. All quantities (except for $\eta _{\Gamma ^1}$) decrease monotonically with refinement. The dominant contributions to the majorant originate from the 2D interfaces, highlighting that MPFA struggles to accurately represent the interface law; this is expected considering that it is a lowest-order method. The right panel of Fig. 6 shows the spatial distribution of the local diffusive errors. Not surprisingly, the largest errors are located around the outlet boundary region, where steep pressure gradients occur.

**Table 3:** Majorant and dimension-based aggregated error estimators for the 3D regular network benchmark from Sect. 7.2

Mesh	$\eta _{\Omega ^3}$	$\eta _{\Omega ^2}$	$\eta _{\Omega ^1}$	$\eta _{\Gamma ^2}$	$\eta _{\Gamma ^1}$	$\eta _{\Gamma ^0}$	$\mathcal {M}^{\oplus }_p=\mathcal {M}^\oplus _{{\boldsymbol{u}}}$
Coarse	5.70e−01	1.39e−02	2.01e−04	1.98e+02	6.47e−01	1.15e−02	1.98e+02
Intermediate	3.84e−01	7.21e−03	1.25e−04	2.90e+01	1.96e+00	2.69e−03	2.90e+01
Fine	3.24e−01	4.04e−03	9.84e−05	6.53e+00	4.06e−01	1.90e−03	6.55e+00

### Production on a fracture network with small features

**Fig. 7:**
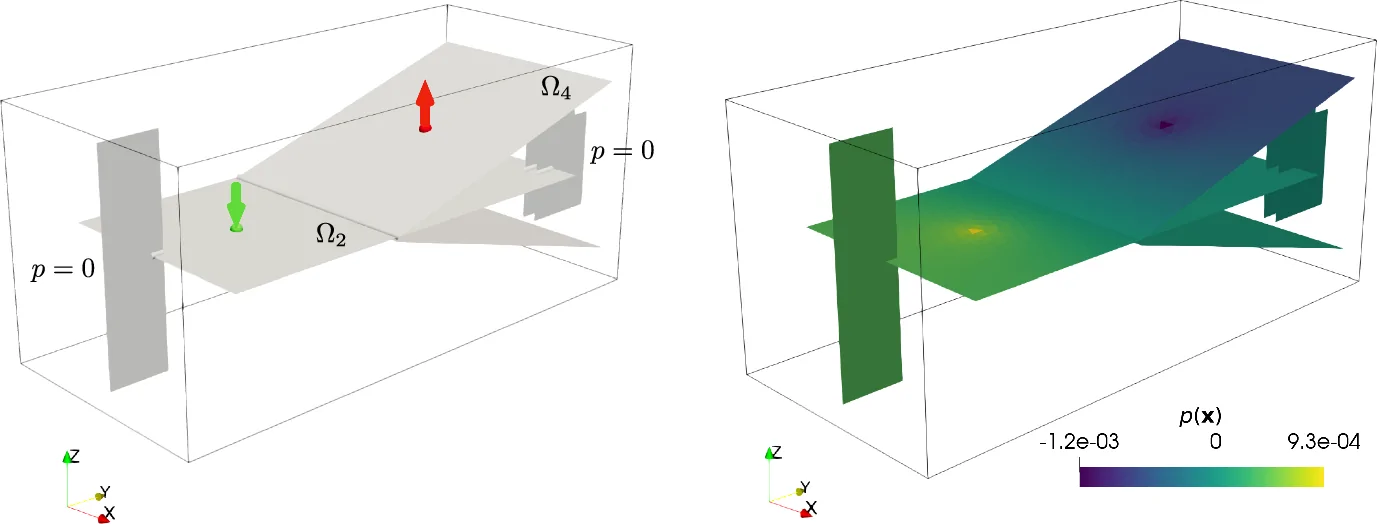
Network with small features. Left: Geometry, boundary conditions, and source/sink locations. Right: Resulting pressure distribution (shown only on 2D subdomains for visualization)

In this numerical example, we consider the setup of Case 3: Network with small features from the 3D verification benchmark [7] to analyze non-matching approximations of an idealized injection/production scenario. To reduce the influence of boundary conditions on the error estimates, we modify the original specification (cf. Section 5.3 of [7]) and impose no-flux boundary conditions on all boundaries except the South and North sides, where we prescribe a constant Dirichlet condition p=0; see the left panel of Fig. 7. We then add an injection well on $\Omega _2$ and a production well on $\Omega _4$, modeled as constant source terms on the well cells:

$$\begin{aligned} f_2(\textbf{x}) = {\left\{ \begin{array}{ll} 0.1, &  \textbf{x}=(0.5, 0.65, 0.5) \\ 0, &  \text {elsewhere} \end{array}\right. } \quad \text {and}\quad f_4(\textbf{x}) = {\left\{ \begin{array}{ll} -0.1, &  \textbf{x}=(0.5, 1.6, 0.675) \\ 0, &  \text {elsewhere} \end{array}\right. } , \end{aligned}$$

where the conditions on $\textbf{x}$ imply the cell containing said coordinate. Following the benchmark, we assign unitary permeability in the matrix $\mathcal {K}=1$ and $\mathcal {K}=1\times 10^{4}$ in the fractures, while the remaining material parameters are taken from Table 5 of [7]. The resulting pressure distribution is shown in the right panel of Fig. 7.

In contrast to the previous numerical example, our goal here is not to perform a full convergence study, but to compare matching and non-matching approximations. As before, the non-matching mD grid is constructed by bisecting the matching mD grid and replacing the finer fracture grids into the coarser mD grid.

**Table 4:** Majorant and dimension-based aggregated error estimators for the 3D fracture network with small features from Sect. 7.3

Approximation	$\eta _{\Omega ^3}$	$\eta _{\Omega ^2}$	$\eta _{\Omega ^1}$	$\eta _{\Gamma ^2}$	$\eta _{\Gamma ^1}$	$\mathcal {M}^{\oplus }_p=\mathcal {M}_{{\boldsymbol{u}}}^\oplus$
Matching	8.08e−04	5.72e−03	3.08e−06	1.22e−01	1.04e−04	1.22e−01
Non-matching	7.89e−04	5.71e−03	2.72e−06	1.24e−01	7.23e−05	1.24e−01

Table 4 shows the aggregated error contributions for both discretizations. The non-matching estimators closely reproduce the matching ones, with differences at most a few percent and a relative deviation of approximately 1.6% in the majorant. The non-matching approximation yields slightly smaller contributions in all aggregated quantities except for $\eta _{\Gamma ^2}$. This illustrates the subtlety of non-matching refinement in mD geometries: refining fractures without fully refining interfaces may reduce errors in some components while increasing them in others. Moreover, as pointed out in Remark 11, $\mathcal {T}_{\Gamma _j}$ cannot be refined freely without simultaneously refining $\mathcal {T}_{\partial _j\Omega _{\hat{\jmath }}}$, as this may violate inf-sup stability conditions [13, 24]. Finally, it is important to remark that the residual contributions vanish for both approximations due to the local mass-conservative properties of MPFA [20].

**Fig. 8:**
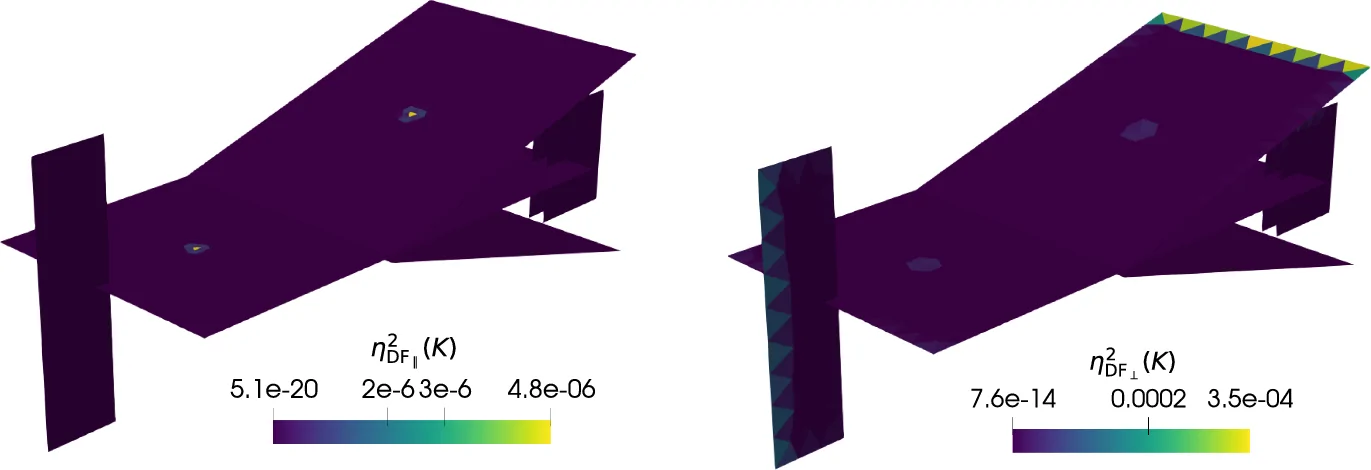
Non-matching local error estimators for the 3D fracture network with small features. Left: 2D diffusive errors on subdomains. Right: 2D diffusive errors on interfaces

Figure 8 shows the spatial distribution of the 2D subdomain and interface non-matching diffusive errors for the non-matching approximation. As expected, the largest subdomain errors occur near the injection and production wells. In contrast, the interface errors are primarily concentrated along the fracture boundaries, which are known to be challenging regions for mD flow discretizations.

## Conclusion

In this article, we have extended the *a posteriori* error estimators developed in [20] for matching grids to the setting of non-matching mD discretizations for elliptic problems, with particular emphasis on single-phase flow in fractured porous media. Using postprocessed MPFA approximations, we verified the validity of the proposed non-matching estimators—and, consequently, of the underlying discrete projection and grid mismatch operators—on synthetic test cases with exact solutions as well as on 3D community benchmark problems featuring complex geometries and realistic material-parameter contrasts. The results confirm that the estimators remain reliable under non-matching refinement, while simultaneously highlighting the delicate interplay between accuracy, refinement choices, and stability requirements in mD settings.

## Data Availability

Code is available for reproduction via the mdnme Python package: jhabriel/non matching estimates: mdnme v0.3, https://doi.org/10.5281/zenodo.17844410.

## Appendix A Proof of Theorem 1

In this section, we provide a complete proof of Theorem 1.

### A.1 Upper bound on the primal variable

Let $p=[p_i]\in H^1_0(\Omega ) + g$ be the solution to primal weak form and let $q=[q_i]\in Q_h^g \subset (H^1_0(\Omega ) + g)$ be an arbitrary potential. Define

$$\begin{aligned} e:=[p_i-q_i]\in H_0^1(\Omega ), \end{aligned}$$

as the primal error. Thus,

A1 $$\begin{aligned} {\left| \hspace{-1.0625pt}\left| \hspace{-1.0625pt}\left| e \right| \hspace{-1.0625pt}\right| \hspace{-1.0625pt}\right| }^2&= \sum _{i\in I} {\left( {\mathcal {K}_i \nabla _i (p_i-q_i)},{\nabla _ie_i}\right) }_{\Omega _i} \nonumber \\&\quad + \sum _{j\in J} {\left( {\kappa _j\left( \mathcal {P}_{\check{\jmath }}^j (p_{\check{\jmath }}- q_{\check{\jmath }})-\mathcal P_{\hat{\jmath }}^j (p_{\hat{\jmath }}- q_{\hat{\jmath }}) \right) },{\mathcal {P}_{\check{\jmath }}^j e_{\check{\jmath }}-\mathcal P_{\hat{\jmath }}^j e_{\hat{\jmath }}}\right) }_{\Gamma _j} \nonumber \\&= \sum _{i\in I} {\left( {\mathcal {K}_i \nabla _i p_i},{\nabla _i(p_i-q_i)}\right) }_{\Omega _i} + \sum _{i\in I} {\left( {-\mathcal {K}_i\nabla _iq_i},{\nabla _i e_i}\right) }_{\Omega _i} \nonumber \\&\quad + \sum _{j\in J} {\left( {\kappa _j\left( \mathcal {P}_{\check{\jmath }}^j p_{\check{\jmath }}- \mathcal P_{\hat{\jmath }}^j p_{\hat{\jmath }}\right) },{\mathcal {P}_{\check{\jmath }}^j e_{\check{\jmath }}-\mathcal P_{\hat{\jmath }}^j e_{\hat{\jmath }}}\right) }_{\Gamma _j} \nonumber \\&\quad + \sum _{j\in J} {\left( {-\kappa _j\left( \mathcal {P}_{\check{\jmath }}^j q_{\check{\jmath }}-\mathcal P_{\hat{\jmath }}^j q_{\hat{\jmath }}\right) },{\mathcal {P}_{\check{\jmath }}^j e_{\check{\jmath }}-\mathcal P_{\hat{\jmath }}^j e_{\hat{\jmath }}}\right) }_{\Gamma _j} \end{aligned}$$

Since $e \in H^1_0(\Omega )$, it may be used as a test function in the weak primal formulation. Hence, the sum of the first and third terms of (A1) coincides with the right-hand side of the weak primal form (Problem 3). We can therefore write the equality

A2 $$\begin{aligned} {\left| \hspace{-1.0625pt}\left| \hspace{-1.0625pt}\left| e \right| \hspace{-1.0625pt}\right| \hspace{-1.0625pt}\right| }^2&= \sum _{i\in I} {\left( {f_i},{e_i}\right) }_{\Omega _i} + \sum _{i\in I} {\left( {-\mathcal {K}_i\nabla _iq_i},{\nabla _ie_i}\right) }_{\Omega _i} \nonumber \\&\quad + \sum _{j\in J} {\left( {-\kappa _j\left( \mathcal {P}_{\check{\jmath }}^j q_{\check{\jmath }}-\mathcal P_{\hat{\jmath }}^j q_{\hat{\jmath }}\right) },{\mathcal {P}_{\check{\jmath }}^j e_{\check{\jmath }}-\mathcal P_{\hat{\jmath }}^j e_{\hat{\jmath }}}\right) }_{\Gamma _j}. \end{aligned}$$

Let us now fix any ${\boldsymbol{v}} = [{\boldsymbol{v}}_{0, i}, \nu _j] \in \textbf{X}_{h,\textrm{LC}}(f)$ and use the mixed-dimensional integration-by-parts formula [1, 20] on the pair $(e, {\boldsymbol{v}}) \in H_0^1(\Omega )\times \textbf{X}_{h,\textrm{LC}}(f)$ (the embedding $\textbf{X}_{h,\textrm{LC}}(f)\subset \textbf{X}$ allows to invoke the mD integration by parts on continuous data) to construct the identity:

A3 $$\begin{aligned}&-\sum _{i\in I} {\left( {\nabla _i \cdot \left( {\boldsymbol{v}}_{0, i} + \textstyle {\sum }_{j\in \check{S}_i} \mathcal {R}^*_j\circ \mathcal {D}_j^{{\hat{\jmath }}} \nu _j\right) - \textstyle {\sum }_{j\in \hat{S}_i}\mathcal {D}_j^{{\check{\jmath }}}\nu _j},{e_i}\right) }_{\Omega _i} \nonumber \\&- \sum _{i\in I} {\left( {{\boldsymbol{v}}_{0, i} + \textstyle {\sum }_{j\in \check{S}_i} \mathcal {R}^*_j\circ \mathcal {D}_j^{{\hat{\jmath }}} \nu _j},{\nabla _i e_i}\right) }_{\Omega _i} - \sum _{j\in J} {\left( {\nu _j},{\mathcal {P}_{\check{\jmath }}^j e_{\check{\jmath }}- \mathcal {P}_{\hat{\jmath }}^j e_{\hat{\jmath }}}\right) }_{\Gamma _j} = 0. \end{aligned}$$

Adding (A3) to (A2), results in the following equality:

A4 $$\begin{aligned} {\left| \hspace{-1.0625pt}\left| \hspace{-1.0625pt}\left| e \right| \hspace{-1.0625pt}\right| \hspace{-1.0625pt}\right| }^2&= \sum _{i\in I} {\left( {f_i - \nabla _i \cdot \left( {\boldsymbol{v}}_{0, i} + \textstyle {\sum }_{j\in \check{S}_i} \mathcal {R}^*_j\circ \mathcal {D}_j^{{\hat{\jmath }}} \nu _j\right) + \textstyle {\sum }_{j\in \hat{S}_i}\mathcal {D}_j^{{\check{\jmath }}}\nu _j},{e_i}\right) }_{\Omega _i} \nonumber \\&\quad + \sum _{i\in I} {\left( {- \left( {\boldsymbol{v}}_{0, i} + \textstyle {\sum }_{j\in \check{S}_i} \mathcal {R}^*_j\circ \mathcal {D}_j^{{\hat{\jmath }}} \nu _j + \mathcal {K}_i\nabla _i q_i\right) },{\nabla _ie_i}\right) }_{\Omega _i} \nonumber \\&\quad + \sum _{j\in J} {\left( {- \left( \nu _j + \kappa _j\left( \mathcal {P}_{\check{\jmath }}^j q_{\check{\jmath }}-\mathcal P_{\hat{\jmath }}^j q_{\hat{\jmath }}\right) \right) },{\mathcal {P}_{\check{\jmath }}^j e_{\check{\jmath }}-\mathcal P_{\hat{\jmath }}^j e_{\hat{\jmath }}}\right) }_{\Gamma _j}. \end{aligned}$$

Using the additive splittings between continuous transfer operators and their discrete realizations,

$$\begin{aligned} \mathcal {D}_j^{\hat{\jmath }}\nu _j&= \widetilde{\mathcal {D}}_j^{\hat{\jmath }}\nu _j + \delta \mathcal {D}_j^{\hat{\jmath }}\nu _j,&\qquad \mathcal {D}_j^{\check{\jmath }}\nu _j&= \widetilde{\mathcal {D}}_j^{\check{\jmath }}\nu _j + \delta \mathcal {D}_j^{\check{\jmath }}\nu _j, \\ \mathcal {P}^j_{\hat{\jmath }}q_{\hat{\jmath }}&= \widetilde{\mathcal {P}}^j_{\hat{\jmath }}q_{\hat{\jmath }}+ \delta \mathcal {P}^j_{\hat{\jmath }}q_{\hat{\jmath }},&\qquad \mathcal {P}^j_{\check{\jmath }}q_{\check{\jmath }}&= \widetilde{\mathcal {P}}^j_{\check{\jmath }}q_{\check{\jmath }}+ \delta \mathcal {P}^j_{\check{\jmath }}q_{\check{\jmath }}, \end{aligned}$$

together with the convention that $\widetilde{\mathcal {R}}^*_j$ acts as the discrete realization of $\mathcal {R}^*_j$ on the matching internal-boundary–bulk coupling (cf. Sect. 4.2), the positive square roots of the positive definite factorization of $\mathcal {K}_i$ and $\kappa _j$, and the additivity of the subdomain and interface inner products over the corresponding non-matching grids, identity (A4) can be written as

A5 $$\begin{aligned} {\left| \hspace{-1.0625pt}\left| \hspace{-1.0625pt}\left| e \right| \hspace{-1.0625pt}\right| \hspace{-1.0625pt}\right| }^2&= \sum _{i\in I} \sum _{K\in \mathcal {T}_{\Omega _i}} \underbrace{{\left( {f_i - \nabla _i \cdot \left( {\boldsymbol{v}}_{0, i} + \textstyle {\sum }_{j\in \check{S}_i} \widetilde{\mathcal {R}}^*_j\circ \widetilde{\mathcal {D}}_j^{{\hat{\jmath }}} \nu _j\right) + \textstyle {\sum }_{j\in \hat{S}_i} \widetilde{\mathcal {D}}_j^{{\check{\jmath }}}\nu _j},{e_i}\right) }_{K}}_{T_1} \nonumber \\&\quad + \sum _{i\in I} \sum _{K\in \mathcal {T}_{\Omega _i}} \underbrace{{\left( {- \mathcal {K}_i^{-1/2}\left( {\boldsymbol{v}}_{0, i} + \textstyle {\sum }_{j\in \check{S}_i} \widetilde{\mathcal {R}}^*_j\circ \widetilde{\mathcal {D}}_j^{{\hat{\jmath }}} \nu _j + \mathcal {K}_i\nabla _i q_i\right) },{\mathcal {K}_i^{1/2}\nabla _ie_i}\right) }_{K}}_{T_2} \nonumber \\&\quad + \sum _{j\in J} \sum _{K\in \mathcal {T}_{\Gamma _j}} \underbrace{{\left( {- \kappa _j^{-1/2}\left( \nu _j + \kappa _j\left( \widetilde{\mathcal {P}}_{\check{\jmath }}^j q_{\check{\jmath }}-\widetilde{\mathcal {P}}_{\hat{\jmath }}^j q_{\hat{\jmath }}\right) \right) },{\kappa _j^{1/2}(\mathcal {P}_{\check{\jmath }}^j e_{\check{\jmath }}-\mathcal {P}_{\hat{\jmath }}^j e_{\hat{\jmath }}) }\right) }_{K}}_{T_3} \nonumber \\&\quad + \sum _{i\in I} \sum _{K\in \mathcal {T}_{\Omega _i}} \underbrace{{\left( {\nabla _i \cdot \textstyle {\sum }_{j\in \check{S}_i} \widetilde{\mathcal {R}}^*_j\circ \delta \mathcal {D}_j^{{\hat{\jmath }}} \nu _j + \textstyle {\sum }_{j\in \hat{S}_i} \delta \mathcal {D}_j^{{\check{\jmath }}}\nu _j},{e_i}\right) }_{K}}_{T_4} \nonumber \\&\quad + \sum _{i\in I} \sum _{K\in \mathcal {T}_{\Omega _i}} \underbrace{{\left( {- \mathcal {K}_i^{-1/2}\textstyle {\sum }_{j\in \check{S}_i} \widetilde{\mathcal {R}}^*_j\circ \delta {\mathcal {D}}_j^{{\hat{\jmath }}} \nu _j},{\mathcal {K}_i^{1/2}\nabla _i e_i}\right) }_{K}}_{T_5} \nonumber \\&\quad + \sum _{j\in J} \sum _{K\in \mathcal {T}_{\Gamma _j}} \underbrace{{\left( {-\kappa _j^{-1/2} \left( \kappa _j\left( \delta \mathcal {P}_{\check{\jmath }}^j q_{\check{\jmath }}-\delta \mathcal {P}_{\hat{\jmath }}^j q_{\hat{\jmath }}\right) \right) },{\kappa _j^{1/2}(\mathcal {P}_{{\check{\jmath }}}^j e_{\check{\jmath }}-\mathcal {P}_{{\hat{\jmath }}}^j e_{\hat{\jmath }}) }\right) }_{K}}_{T_6}. \end{aligned}$$

For the sake of compactness, in the following, we shall use

A6 $$\begin{aligned} \sum _{i,K} \bullet =\sum _{i\in I} \sum _{K\in \mathcal {T}_{\Omega _i}} \bullet , \qquad \sum _{j,K} \bullet =\sum _{j\in J} \sum _{K\in \mathcal {T}_{\Gamma _j}} \bullet . \end{aligned}$$

We now bound each of the six terms cellwise.

*Bound on* $T_1$ *.* The flux ${\boldsymbol{v}}\in \textbf{X}_{h,\textrm{LC}}(f)$ satisfies the local mass-conservation property ${\left( {\widetilde{r}_i},{1}\right) }_K = 0$ for every $K\in \mathcal {T}_{\Omega _i}$ (cf. Definition 7), where

$$\begin{aligned} \widetilde{r}_i := f_i - \nabla _i\bullet \Bigl ({\boldsymbol{v}}_{0,i} + \textstyle {\sum }_{j\in \check{S}_i}\widetilde{\mathcal {R}}^*_j\circ \widetilde{\mathcal {D}}_j^{{\hat{\jmath }}}\nu _j\Bigr ) + \textstyle {\sum }_{j\in \hat{S}_i}\widetilde{\mathcal {D}}_j^{{\check{\jmath }}}\nu _j \end{aligned}$$

denotes the discrete residual on *K*. Subtracting the cell mean $\overline{e_i}^K \in \mathbb {R}$ of $e_i$ on *K* leaves the term unchanged, and the Poincaré–Wirtinger inequality on the convex simplex *K* together with the ellipticity bound $\left\Vert \nabla _i e_i\right\Vert _K \le c_{\mathcal {K}_i,K}^{-1}\left\Vert \mathcal {K}_i^{1/2}\nabla _i e_i\right\Vert _K$ from Assumption 2 yield

A7 $$\begin{aligned} |T_1|&= \left|{\left( {\widetilde{r}_i},{e_i - \overline{e_i}^K}\right) }_K\right| \le \left\Vert \widetilde{r}_i\right\Vert _K \cdot \frac{h_K}{\pi }\left\Vert \nabla _i e_i\right\Vert _K \nonumber \\&\le \frac{h_K}{\pi c_{\mathcal {K}_i,K}}\left\Vert \widetilde{r}_i\right\Vert _K \cdot \left\Vert \mathcal {K}_i^{1/2}\nabla _i e_i\right\Vert _K = \eta _{\textrm{NM, R},K}\,\left\Vert \mathcal {K}_i^{1/2}\nabla _i e_i\right\Vert _K. \end{aligned}$$

*Bound on* $T_4$ *.* Define the grid-mismatch residual

$$\begin{aligned} \rho _i := \nabla _i\cdot \textstyle {\sum }_{j\in \check{S}_i}\widetilde{\mathcal {R}}^*_j\circ \delta \mathcal {D}_j^{{\hat{\jmath }}}\nu _j + \textstyle {\sum }_{j\in \hat{S}_i}\delta \mathcal {D}_j^{{\check{\jmath }}}\nu _j, \end{aligned}$$

so that $T_4 = {\left( {\rho _i},{e_i}\right) }_K$. By the cellwise mass-defect property of the discrete grid-mismatch operators (cf. Lemma 3), ${\left( {\rho _i},{1}\right) }_K = 0$ for every $K\in \mathcal {T}_{\Omega _i}$. The same Poincaré–Wirtinger argument as for $T_1$ then yields

A8 $$\begin{aligned} |T_4| \le \frac{h_K}{\pi c_{\mathcal {K}_i,K}}\left\Vert \rho _i\right\Vert _K \cdot \left\Vert \mathcal {K}_i^{1/2}\nabla _i e_i\right\Vert _K = \eta _{\textrm{GM},\textrm{R},K}\,\left\Vert \mathcal {K}_i^{1/2}\nabla _i e_i\right\Vert _K. \end{aligned}$$

*Bounds on* $T_2$ *and* $T_5$ *.* Direct application of the Cauchy–Schwarz inequality gives

A9 $$\begin{aligned} |T_2|&\le \left\Vert \mathcal {K}_i^{-1/2}\Bigl ({\boldsymbol{v}}_{0,i} + \textstyle {\sum }_{j\in \check{S}_i}\widetilde{\mathcal {R}}^*_j\circ \widetilde{\mathcal {D}}_j^{{\hat{\jmath }}}\nu _j\Bigr ) + \mathcal {K}_i^{1/2}\nabla _i q_i\right\Vert _K \cdot \left\Vert \mathcal {K}_i^{1/2}\nabla _i e_i\right\Vert _K \nonumber \\&= \eta _{\textrm{NM, DF}_\parallel ,K}\,\left\Vert \mathcal {K}_i^{1/2}\nabla _i e_i\right\Vert _K, \end{aligned}$$

A10 $$\begin{aligned} |T_5|&\le \left\Vert \mathcal {K}_i^{-1/2}\textstyle {\sum }_{j\in \check{S}_i}\widetilde{\mathcal {R}}^*_j\circ \delta \mathcal {D}_j^{{\hat{\jmath }}}\nu _j\right\Vert _K \cdot \left\Vert \mathcal {K}_i^{1/2}\nabla _i e_i\right\Vert _K = \eta _{\textrm{GM},\textrm{DF}_\parallel ,K}\,\left\Vert \mathcal {K}_i^{1/2}\nabla _i e_i\right\Vert _K. \end{aligned}$$

*Bounds on* $T_3$ *and* $T_6$ *.* Analogously, on each $K\in \mathcal {T}_{\Gamma _j}$,

A11 $$\begin{aligned} |T_3|&\le \left\Vert \kappa _j^{-1/2}\nu _j + \kappa _j^{1/2}\bigl (\widetilde{\mathcal {P}}_{\check{\jmath }}^j q_{\check{\jmath }}- \widetilde{\mathcal {P}}_{\hat{\jmath }}^j q_{\hat{\jmath }}\bigr )\right\Vert _K \cdot \left\Vert \kappa _j^{1/2}\bigl (\mathcal {P}_{\check{\jmath }}^j e_{\check{\jmath }}- \mathcal {P}_{\hat{\jmath }}^j e_{\hat{\jmath }}\bigr )\right\Vert _K \nonumber \\&= \eta _{\textrm{NM, DF}_\perp ,K}\,\left\Vert \kappa _j^{1/2}\bigl (\mathcal {P}_{\check{\jmath }}^j e_{\check{\jmath }}- \mathcal {P}_{\hat{\jmath }}^j e_{\hat{\jmath }}\bigr )\right\Vert _K, \end{aligned}$$

A12 $$\begin{aligned} |T_6|&\le \left\Vert \kappa _j^{1/2}\bigl (\delta \mathcal {P}_{\check{\jmath }}^j q_{\check{\jmath }}- \delta \mathcal {P}_{\hat{\jmath }}^j q_{\hat{\jmath }}\bigr )\right\Vert _K \cdot \left\Vert \kappa _j^{1/2}\bigl (\mathcal {P}_{\check{\jmath }}^j e_{\check{\jmath }}- \mathcal {P}_{\hat{\jmath }}^j e_{\hat{\jmath }}\bigr )\right\Vert _K \nonumber \\&= \eta _{\textrm{GM},\textrm{DF}_\perp ,K}\,\left\Vert \kappa _j^{1/2}\bigl (\mathcal {P}_{\check{\jmath }}^j e_{\check{\jmath }}- \mathcal {P}_{\hat{\jmath }}^j e_{\hat{\jmath }}\bigr )\right\Vert _K. \end{aligned}$$

Setting

$$\begin{aligned} a_K := \left\Vert \mathcal {K}_i^{1/2}\nabla _i e_i\right\Vert _K\ (K\in \mathcal {T}_{\Omega _i}), \qquad b_K := \left\Vert \kappa _j^{1/2}\bigl (\mathcal {P}_{\check{\jmath }}^j e_{\check{\jmath }}- \mathcal {P}_{\hat{\jmath }}^j e_{\hat{\jmath }}\bigr )\right\Vert _K\ (K\in \mathcal {T}_{\Gamma _j}), \end{aligned}$$

the definition of ${\left| \hspace{-1.0625pt}\left| \hspace{-1.0625pt}\left| \cdot \right| \hspace{-1.0625pt}\right| \hspace{-1.0625pt}\right| }$ in Sect. 3.3 gives the identity $\sum _{i,K} a_K^2 + \sum _{j,K} b_K^2 = {\left| \hspace{-1.0625pt}\left| \hspace{-1.0625pt}\left| e \right| \hspace{-1.0625pt}\right| \hspace{-1.0625pt}\right| }^2$. Combining the cellwise bounds (A7)–(A12) in (A5) and grouping the right-hand sides by their dependence on $a_K$ or $b_K$ yields

A13 $$\begin{aligned} {\left| \hspace{-1.0625pt}\left| \hspace{-1.0625pt}\left| e \right| \hspace{-1.0625pt}\right| \hspace{-1.0625pt}\right| }^2&\le \sum _{i,K} \bigl (\eta _{\textrm{NM,R},K} + \eta _{\textrm{GM},\textrm{R},K} + \eta _{\textrm{NM, DF}_\parallel ,K} + \eta _{\textrm{GM},\textrm{DF}_\parallel ,K}\bigr )\,a_K \nonumber \\&\quad + \sum _{j,K} \bigl (\eta _{\textrm{NM, DF}_\perp ,K} + \eta _{\textrm{GM},\textrm{DF}_\perp ,K}\bigr )\,b_K. \end{aligned}$$

We bound each of the four global indicators of (32) by Cauchy–Schwarz. For the NM residual contributions, $\sum _{i,K}a_K^2 \le {\left| \hspace{-1.0625pt}\left| \hspace{-1.0625pt}\left| e \right| \hspace{-1.0625pt}\right| \hspace{-1.0625pt}\right| }^2$ gives

$$\begin{aligned} \sum _{i,K} \eta _{\textrm{NM, R},K}\,a_K \le \eta _{\textrm{NM,R}}({\boldsymbol{v}},f)\,{\left| \hspace{-1.0625pt}\left| \hspace{-1.0625pt}\left| e \right| \hspace{-1.0625pt}\right| \hspace{-1.0625pt}\right| }, \qquad \sum _{i,K} \eta _{\textrm{GM},\textrm{R},K}\,a_K \le \eta _{\textrm{GM,R}}({\boldsymbol{v}})\,{\left| \hspace{-1.0625pt}\left| \hspace{-1.0625pt}\left| e \right| \hspace{-1.0625pt}\right| \hspace{-1.0625pt}\right| }. \end{aligned}$$

For the NM diffusive contributions we apply Cauchy–Schwarz *jointly* on the bulk and interface index sets to the $\eta _{\textrm{NM,DF}_\parallel ,K}\sqcup \eta _{\textrm{NM,DF}_\perp ,K}$ and $a_K\sqcup b_K$ vectors:

$$\begin{aligned} \sum _{i,K}\eta _{\textrm{NM,DF}_\parallel ,K}\,a_K + \sum _{j,K}\eta _{\textrm{NM,DF}_\perp ,K}\,b_K&\le \sqrt{\eta _{\textrm{NM,DF}_\parallel }^2 + \eta _{\textrm{NM,DF}_\perp }^2}\,\sqrt{\sum _{i,K}a_K^2 + \sum _{j,K}b_K^2} \\&= \eta _{\textrm{NM,DF}}(q,{\boldsymbol{v}})\,{\left| \hspace{-1.0625pt}\left| \hspace{-1.0625pt}\left| e \right| \hspace{-1.0625pt}\right| \hspace{-1.0625pt}\right| }, \end{aligned}$$

by definition (33c), and analogously

$$\begin{aligned} \sum _{i,K}\eta _{\textrm{GM},\textrm{DF}_\parallel ,K}\,a_K + \sum _{j,K}\eta _{\textrm{GM},\textrm{DF}_\perp ,K}\,b_K \le \eta _{\textrm{GM,DF}}(q,{\boldsymbol{v}})\,{\left| \hspace{-1.0625pt}\left| \hspace{-1.0625pt}\left| e \right| \hspace{-1.0625pt}\right| \hspace{-1.0625pt}\right| }, \end{aligned}$$

by definition (33d). Substituting the four bounds back into (A13) and dividing by ${\left| \hspace{-1.0625pt}\left| \hspace{-1.0625pt}\left| e \right| \hspace{-1.0625pt}\right| \hspace{-1.0625pt}\right| }$ (the case ${\left| \hspace{-1.0625pt}\left| \hspace{-1.0625pt}\left| e \right| \hspace{-1.0625pt}\right| \hspace{-1.0625pt}\right| }=0$ being trivial) yields

$$\begin{aligned} {\left| \hspace{-1.0625pt}\left| \hspace{-1.0625pt}\left| p-q \right| \hspace{-1.0625pt}\right| \hspace{-1.0625pt}\right| } \le \eta _{\textrm{NM,R}}({\boldsymbol{v}},f) + \eta _{\textrm{GM,R}}({\boldsymbol{v}}) + \eta _{\textrm{NM,DF}}(q,{\boldsymbol{v}}) + \eta _{\textrm{GM,DF}}(q,{\boldsymbol{v}}) = \mathcal {M}(q,{\boldsymbol{v}},f) =: \mathcal {M}^{\oplus }_p, \end{aligned}$$

which is the upper bound for the primal variable in part (i) of Theorem 1. □

### A.2 Upper bound on the dual variable

We mimic the proof of part (b) of [20, Theorem 5.1, Appendix C], with the additional grid-mismatch contributions arising from the splitting $\mathcal {D} = \widetilde{\mathcal {D}} + \delta \mathcal {D}$ and $\mathcal {P} = \widetilde{\mathcal {P}} + \delta \mathcal {P}$ on the non-matching coupling triplets. Let ${\boldsymbol{u}}=[{\boldsymbol{u}}_{0,i}, \lambda _j]\in \textbf{X}$ be the solution of the dual weak form (Problem 4), and let $(q,{\boldsymbol{v}})\in Q_h^g\times \textbf{X}_{h,\textrm{LC}}(f)$ be an arbitrary admissible pair. Define the bulk-lifted fluxes

$$\begin{aligned} {\boldsymbol{u}}_i := {\boldsymbol{u}}_{0,i} + \textstyle {\sum }_{j\in \check{S}_i}\mathcal {R}^*_j\circ \mathcal {D}_j^{{\hat{\jmath }}}\lambda _j, \qquad {\boldsymbol{v}}_i := {\boldsymbol{v}}_{0,i} + \textstyle {\sum }_{j\in \check{S}_i}\mathcal {R}^*_j\circ \mathcal {D}_j^{{\hat{\jmath }}}\nu _j, \end{aligned}$$

both regarded as elements of $\textbf{V}_i$ via the continuous lift $\mathcal {R}^*_j$, in agreement with the definition of the dual energy norm ${\left| \hspace{-1.0625pt}\left| \hspace{-1.0625pt}\left| \,\cdot \, \right| \hspace{-1.0625pt}\right| \hspace{-1.0625pt}\right| }_*$ in Sect. 3.3.

Adding the squares of the primal and dual error norms,

A14 $$\begin{aligned}&{\left| \hspace{-1.0625pt}\left| \hspace{-1.0625pt}\left| p-q \right| \hspace{-1.0625pt}\right| \hspace{-1.0625pt}\right| }^2 + {\left| \hspace{-1.0625pt}\left| \hspace{-1.0625pt}\left| {\boldsymbol{u}}-{\boldsymbol{v}} \right| \hspace{-1.0625pt}\right| \hspace{-1.0625pt}\right| }_*^2 = \sum _{i\in I}{\left( {\mathcal {K}_i\nabla _i(p_i-q_i)},{\nabla _i(p_i-q_i)}\right) }_{\Omega _i} \nonumber \\&\quad + \sum _{j\in J}{\left( {\kappa _j\bigl (\mathcal {P}_{\check{\jmath }}^j(p_{\check{\jmath }}-q_{\check{\jmath }})-\mathcal {P}_{\hat{\jmath }}^j(p_{\hat{\jmath }}-q_{\hat{\jmath }})\bigr )},{\mathcal {P}_{\check{\jmath }}^j(p_{\check{\jmath }}-q_{\check{\jmath }})-\mathcal {P}_{\hat{\jmath }}^j(p_{\hat{\jmath }}-q_{\hat{\jmath }})}\right) }_{\Gamma _j} \nonumber \\&\quad + \sum _{i\in I}{\left( {\mathcal {K}_i^{-1}({\boldsymbol{u}}_i-{\boldsymbol{v}}_i)},{{\boldsymbol{u}}_i-{\boldsymbol{v}}_i}\right) }_{\Omega _i} + \sum _{j\in J}{\left( {\kappa _j^{-1}(\lambda _j-\nu _j)},{\lambda _j-\nu _j}\right) }_{\Gamma _j}. \end{aligned}$$

The exact pair $({\boldsymbol{u}},\lambda ,p)$ inherits from the dual weak form the constitutive relations

$$\begin{aligned} \mathcal {K}_i^{-1}{\boldsymbol{u}}_i = -\nabla _i p_i \text { in } \Omega _i, \qquad \kappa _j^{-1}\lambda _j = -\bigl (\mathcal {P}_{\check{\jmath }}^j p_{\check{\jmath }}- \mathcal {P}_{\hat{\jmath }}^j p_{\hat{\jmath }}\bigr ) \text { on } \Gamma _j, \end{aligned}$$

which allow the bulk and interface diffusive contributions of (A14) to be rewritten as

$$\begin{aligned} {\left( {\mathcal {K}_i\nabla _i(p_i-q_i)},{\nabla _i(p_i-q_i)}\right) }_{\Omega _i}&= \left\Vert \mathcal {K}_i^{-1/2}({\boldsymbol{u}}_i + \mathcal {K}_i\nabla _i q_i)\right\Vert _{\Omega _i}^2, \\ {\left( {\kappa _j J(p-q)},{J(p-q)}\right) }_{\Gamma _j}&= \left\Vert \kappa _j^{-1/2}(\lambda _j + \kappa _j J(q))\right\Vert _{\Gamma _j}^2, \end{aligned}$$

where we abbreviated $J(\,\cdot \,):= \mathcal {P}_{\check{\jmath }}^j(\,\cdot \,)_{\check{\jmath }}- \mathcal {P}_{\hat{\jmath }}^j(\,\cdot \,)_{\hat{\jmath }}$. Decomposing ${\boldsymbol{u}}_i + \mathcal {K}_i\nabla _i q_i = ({\boldsymbol{u}}_i-{\boldsymbol{v}}_i) + ({\boldsymbol{v}}_i + \mathcal {K}_i\nabla _i q_i)$ and analogously for the interface piece, expanding the squares, and adding the ${\left| \hspace{-1.0625pt}\left| \hspace{-1.0625pt}\left| {\boldsymbol{u}}-{\boldsymbol{v}} \right| \hspace{-1.0625pt}\right| \hspace{-1.0625pt}\right| }_*^2$ contributions of (A14) yields the algebraic identity (cf. [20, Eq. (C.4)])

A15 $$\begin{aligned} {\left| \hspace{-1.0625pt}\left| \hspace{-1.0625pt}\left| p-q \right| \hspace{-1.0625pt}\right| \hspace{-1.0625pt}\right| }^2 + {\left| \hspace{-1.0625pt}\left| \hspace{-1.0625pt}\left| {\boldsymbol{u}}-{\boldsymbol{v}} \right| \hspace{-1.0625pt}\right| \hspace{-1.0625pt}\right| }_*^2 = {\left| \hspace{-1.0625pt}\left| \hspace{-1.0625pt}\left| {\boldsymbol{v}}+\mathcal {K}\nabla q \right| \hspace{-1.0625pt}\right| \hspace{-1.0625pt}\right| }_*^2 + 2\,\Theta (p-q,{\boldsymbol{u}}-{\boldsymbol{v}}), \end{aligned}$$

where the diffusive functional and the cross term are

$$\begin{aligned} {\left| \hspace{-1.0625pt}\left| \hspace{-1.0625pt}\left| {\boldsymbol{v}}+\mathcal {K}\nabla q \right| \hspace{-1.0625pt}\right| \hspace{-1.0625pt}\right| }_*^2&:= \sum _{i\in I}\left\Vert \mathcal {K}_i^{-1/2}({\boldsymbol{v}}_i+\mathcal {K}_i\nabla _i q_i)\right\Vert _{\Omega _i}^2 + \sum _{j\in J}\left\Vert \kappa _j^{-1/2}(\nu _j+\kappa _j J(q))\right\Vert _{\Gamma _j}^2, \\ \Theta (p-q,{\boldsymbol{u}}-{\boldsymbol{v}})&:= -\sum _{i\in I}{\left( {{\boldsymbol{u}}_i-{\boldsymbol{v}}_i},{\nabla _i(p_i-q_i)}\right) }_{\Omega _i} - \sum _{j\in J}{\left( {\lambda _j-\nu _j},{J(p-q)}\right) }_{\Gamma _j}. \end{aligned}$$

Applying the mD integration-by-parts formula [1] to the pair $(p-q,{\boldsymbol{u}}-{\boldsymbol{v}})\in H_0^1(\Omega )\times \textbf{X}$ (in the same form as (A3)) and substituting on Θ,

$$\begin{aligned} \Theta (p-q,{\boldsymbol{u}}-{\boldsymbol{v}}) = \sum _{i\in I}{\left( {\nabla _i\cdot ({\boldsymbol{u}}_i-{\boldsymbol{v}}_i) - \textstyle {\sum }_{j\in \hat{S}_i}\mathcal {D}_j^{{\check{\jmath }}}(\lambda _j-\nu _j)},{p_i-q_i}\right) }_{\Omega _i}. \end{aligned}$$

The continuous mass-conservation identity $\nabla _i\cdot {\boldsymbol{u}}_i - \textstyle {\sum }_{j\in \hat{S}_i}\mathcal {D}_j^{{\check{\jmath }}}\lambda _j = f_i$, inherited from the second equation of Problem 4, then reduces the cross term to

$$\begin{aligned} \Theta (p-q,{\boldsymbol{u}}-{\boldsymbol{v}}) = \sum _{i\in I}{\left( {r_i},{p_i-q_i}\right) }_{\Omega _i}, \qquad r_i := f_i - \nabla _i\cdot {\boldsymbol{v}}_i + \textstyle {\sum }_{j\in \hat{S}_i}\mathcal {D}_j^{{\check{\jmath }}}\nu _j. \end{aligned}$$

Splitting $r_i = \widetilde{r}_i + \rho _i$ as in the primal proof, the discrete part $\widetilde{r}_i$ has zero cell mean by Definition 7 and the grid-mismatch part $\rho _i$ has zero cell mean by Lemma 3. Subtracting the cell mean $\overline{(p-q)_i}^K$ on every $K\in \mathcal {T}_{\Omega _i}$ and applying the Poincaré–Wirtinger inequality together with the ellipticity bound from Assumption 2 (exactly as in the bound for $T_1$ and $T_4$ in Appendix A.1), then summing cellwise via Cauchy–Schwarz on each indicator separately,

$$\begin{aligned} \left|\Theta (p-q,{\boldsymbol{u}}-{\boldsymbol{v}})\right|&\le \bigl (\eta _{\textrm{NM,R}}({\boldsymbol{v}},f) + \eta _{\textrm{GM,R}}({\boldsymbol{v}})\bigr )\,\left\Vert \mathcal {K}^{1/2}\nabla (p-q)\right\Vert _\Omega \\&\le \bigl (\eta _{\textrm{NM,R}}({\boldsymbol{v}},f) + \eta _{\textrm{GM,R}}({\boldsymbol{v}})\bigr )\,{\left| \hspace{-1.0625pt}\left| \hspace{-1.0625pt}\left| p-q \right| \hspace{-1.0625pt}\right| \hspace{-1.0625pt}\right| }, \end{aligned}$$

where the second inequality uses $\left\Vert \mathcal {K}^{1/2}\nabla (p-q)\right\Vert _\Omega \le {\left| \hspace{-1.0625pt}\left| \hspace{-1.0625pt}\left| p-q \right| \hspace{-1.0625pt}\right| \hspace{-1.0625pt}\right| }$ from the definition of the energy norm ${\left| \hspace{-1.0625pt}\left| \hspace{-1.0625pt}\left| \cdot \right| \hspace{-1.0625pt}\right| \hspace{-1.0625pt}\right| }$.

By the Young inequality $2ab \le a^2 + b^2$ applied to $a = \eta _{\textrm{NM,R}}({\boldsymbol{v}},f)+\eta _{\textrm{GM,R}}({\boldsymbol{v}})$ and $b = {\left| \hspace{-1.0625pt}\left| \hspace{-1.0625pt}\left| p-q \right| \hspace{-1.0625pt}\right| \hspace{-1.0625pt}\right| }$,

$$\begin{aligned} 2\left|\Theta (p-q,{\boldsymbol{u}}-{\boldsymbol{v}})\right| \le \bigl (\eta _{\textrm{NM,R}}({\boldsymbol{v}},f) + \eta _{\textrm{GM,R}}({\boldsymbol{v}})\bigr )^2 + {\left| \hspace{-1.0625pt}\left| \hspace{-1.0625pt}\left| p-q \right| \hspace{-1.0625pt}\right| \hspace{-1.0625pt}\right| }^2. \end{aligned}$$

Substituting into (A15) and cancelling ${\left| \hspace{-1.0625pt}\left| \hspace{-1.0625pt}\left| p-q \right| \hspace{-1.0625pt}\right| \hspace{-1.0625pt}\right| }^2$ from both sides yields

A16 $$\begin{aligned} {\left| \hspace{-1.0625pt}\left| \hspace{-1.0625pt}\left| {\boldsymbol{u}}-{\boldsymbol{v}} \right| \hspace{-1.0625pt}\right| \hspace{-1.0625pt}\right| }_*^2 \le {\left| \hspace{-1.0625pt}\left| \hspace{-1.0625pt}\left| {\boldsymbol{v}}+\mathcal {K}\nabla q \right| \hspace{-1.0625pt}\right| \hspace{-1.0625pt}\right| }_*^2 + \bigl (\eta _{\textrm{NM,R}}({\boldsymbol{v}},f) + \eta _{\textrm{GM,R}}({\boldsymbol{v}})\bigr )^2. \end{aligned}$$

Taking square roots and using $\sqrt{a^2+b^2}\le a+b$ for a,b≥0,

A17 $$\begin{aligned} {\left| \hspace{-1.0625pt}\left| \hspace{-1.0625pt}\left| {\boldsymbol{u}}-{\boldsymbol{v}} \right| \hspace{-1.0625pt}\right| \hspace{-1.0625pt}\right| }_* \le {\left| \hspace{-1.0625pt}\left| \hspace{-1.0625pt}\left| {\boldsymbol{v}}+\mathcal {K}\nabla q \right| \hspace{-1.0625pt}\right| \hspace{-1.0625pt}\right| }_* + \eta _{\textrm{NM,R}}({\boldsymbol{v}},f) + \eta _{\textrm{GM,R}}({\boldsymbol{v}}). \end{aligned}$$

It remains to express ${\left| \hspace{-1.0625pt}\left| \hspace{-1.0625pt}\left| {\boldsymbol{v}}+\mathcal {K}\nabla q \right| \hspace{-1.0625pt}\right| \hspace{-1.0625pt}\right| }_*$ in terms of the diffusive indicators of Theorem 1. Splitting the continuous transfer operators into their discrete realizations and the corresponding defect operators (Definition 5, with the lift convention (27) on $\widetilde{\mathcal {R}}^*_j$),

$$\begin{aligned} \mathcal {K}_i^{-1/2}({\boldsymbol{v}}_i + \mathcal {K}_i\nabla _i q_i)&= \underbrace{\mathcal {K}_i^{-1/2}\Bigl ({\boldsymbol{v}}_{0,i} + \textstyle {\sum }_{j\in \check{S}_i}\widetilde{\mathcal {R}}^*_j\circ \widetilde{\mathcal {D}}_j^{{\hat{\jmath }}}\nu _j\Bigr ) + \mathcal {K}_i^{1/2}\nabla _i q_i}_{\eta _{\textrm{NM,DF}_\parallel ,K}\text { pointwise on }K} \\&\quad + \underbrace{\mathcal {K}_i^{-1/2}\textstyle {\sum }_{j\in \check{S}_i}\widetilde{\mathcal {R}}^*_j\circ \delta \mathcal {D}_j^{{\hat{\jmath }}}\nu _j}_{\eta _{\textrm{GM},\textrm{DF}_\parallel ,K}\text { pointwise on }K}, \\ \kappa _j^{-1/2}(\nu _j + \kappa _j J(q))&= \underbrace{\kappa _j^{-1/2}\nu _j + \kappa _j^{1/2}\bigl (\widetilde{\mathcal {P}}_{\check{\jmath }}^j q_{\check{\jmath }}- \widetilde{\mathcal {P}}_{\hat{\jmath }}^j q_{\hat{\jmath }}\bigr )}_{\eta _{\textrm{NM,DF}_\perp ,K}\text { pointwise on }K} + \underbrace{\kappa _j^{1/2}\bigl (\delta \mathcal {P}_{\check{\jmath }}^j q_{\check{\jmath }}- \delta \mathcal {P}_{\hat{\jmath }}^j q_{\hat{\jmath }}\bigr )}_{\eta _{\textrm{GM},\textrm{DF}_\perp ,K}\text { pointwise on }K}. \end{aligned}$$

Applying the triangle inequality on the product space $L^2(\Omega ) \times L^2(\Gamma )$ to the sum of these two contributions yields

$$\begin{aligned} {\left| \hspace{-1.0625pt}\left| \hspace{-1.0625pt}\left| {\boldsymbol{v}}+\mathcal {K}\nabla q \right| \hspace{-1.0625pt}\right| \hspace{-1.0625pt}\right| }_*&\le \sqrt{\sum _{i, K}\eta _{\textrm{NM,DF}_\parallel ,K}^2 + \sum _{j, K} \eta _{\textrm{NM,DF}_\perp ,K}^2} \\&\quad + \sqrt{\sum _{i, K} \eta _{\textrm{GM},\textrm{DF}_\parallel ,K}^2 + \sum _{j, K} \eta _{\textrm{GM},\textrm{DF}_\perp ,K}^2} \\&= \eta _{\textrm{NM,DF}}(q,{\boldsymbol{v}}) + \eta _{\textrm{GM,DF}}(q,{\boldsymbol{v}}), \end{aligned}$$

by the global indicator definitions (33c) and (33d). Substituting back into (A17),

$$\begin{aligned} {\left| \hspace{-1.0625pt}\left| \hspace{-1.0625pt}\left| {\boldsymbol{u}}-{\boldsymbol{v}} \right| \hspace{-1.0625pt}\right| \hspace{-1.0625pt}\right| }_*\le &   \eta _{\textrm{NM,DF}}(q,{\boldsymbol{v}}) + \eta _{\textrm{GM,DF}}(q,{\boldsymbol{v}}) + \eta _{\textrm{NM,R}}({\boldsymbol{v}},f) + \eta _{\textrm{GM,R}}({\boldsymbol{v}}) \\= &   \mathcal {M}(q,{\boldsymbol{v}},f) =: \mathcal {M}^{\oplus }_{{\boldsymbol{u}}}, \end{aligned}$$

which is the upper bound for the dual variable in part (ii) of Theorem 1. □

## Code Availability

Code is available for reproduction via the mdnme Python package [60].

## Declarations

### Usage of Artificial Intelligence

Parts of the software implementation were developed with the assistance of Claude Code powered by Claude Sonnet 4.6. ChatGPT 5.5 was used to improve readability of selected parts of the manuscript.
